# Au@Nb@H_*x*_K_1-*x*_NbO_3_ nanopeapods with near-infrared active plasmonic hot-electron injection for water splitting

**DOI:** 10.1038/s41467-017-02676-w

**Published:** 2018-01-16

**Authors:** Ying-Chu Chen, Yu-Kuei Hsu, Radian Popescu, Dagmar Gerthsen, Yan-Gu Lin, Claus Feldmann

**Affiliations:** 1 Institut für Anorganische Chemie, Karlsruhe Institute of Technology (KIT), Engesserstraße 15, Karlsruhe, D-76131 Germany; 2 Department of Opto-Electronic Engineering, National Dong Hwa University, Hualien, 97401 Taiwan; 3 Laboratorium für Elektronenmikroskopie, Karlsruhe Institute of Technology (KIT), Engesserstraße 7, Karlsruhe, D-76131 Germany; 4 National Synchrotron Radiation Research Center, Hsinchu, 30076 Taiwan

## Abstract

Full-spectrum utilization of diffusive solar energy by a photocatalyst for environmental remediation and fuel generation has long been pursued. In contrast to tremendous efforts in the UV-to-VIS light regime of the solar spectrum, the NIR and IR areas have been barely addressed although they represent about 50% of the solar flux. Here we put forward a biomimetic photocatalyst blueprint that emulates the growth pattern of a natural plant—a peapod—to address this issue. This design is exemplified via unidirectionally seeding core-shell Au@Nb nanoparticles in the cavity of semiconducting H_*x*_K_1−*x*_NbO_3_ nanoscrolls. The biomimicry of this nanopeapod (NPP) configuration promotes near-field plasmon–plasmon coupling between bimetallic Au@Nb nanoantennas (the peas), endowing the UV-active H_*x*_K_1−*x*_NbO_3_ semiconductor (the pods) with strong VIS and NIR light harvesting abilities. Moreover, the characteristic 3D metal-semiconductor junction of the Au@Nb@H_*x*_K_1−*x*_NbO_3_ NPPs favors the transfer of plasmonic hot carriers to trigger dye photodegradation and water photoelectrolysis as proofs-of-concept. Such broadband solar spectral response renders the Au@Nb@H_*x*_K_1−*x*_NbO_3_ NPPs highly promising for widespread photoactive devices.

## Abstract (web summary)

Although near-infrared light makes up a large portion of the solar spectrum, harvesting it for photocatalytic applications remains challenging. Here the authors deposit unidirectional Au@Nb core-shell nanoparticles into tubular H_*x*_K_1–*x*_NbO_3_ nanoscrolls and report cooperative full solar spectrum absorption.

## Introduction

For millennia, nature routinely has fed humanity with useful energy in either food supplies or carbon-based fuels by solar-to-chemical conversion, namely photosynthesis using sunlight^1–4^. In particular, these carbohydrates are produced from water and carbon dioxide. The sustainability and environmental benignity in the context of photosynthesis have inspired scientists and engineers to emulate so-called artificial photosynthesis or photocatalysis using man-made materials^5–12^. One of the major endeavors in the field addresses the sunlight-harvesting ability of the photocatalyst, preferentially ranging from ultraviolet (UV) to visible (VIS) light—accounting for nearly 50% of the solar spectrum—to photogenerate electron/hole pairs (e^−^/h^+^) for fuel production^13–17^. Although near-infrared (NIR) light is responsible for an additional 50% of the solar flux, quite to our surprise, further exploitation on its charge-carrier-generation efficacy is currently in its infancy^18–21^. In the light of its enormous percentage in sunlight, charge-carrier generation in the NIR spectral region is of obvious significance.

To this end, coupling of a photocatalyst with plasmonic nanoantennas stands out since the size and shape of metallic nanostructures can be readily used to tailor the light-harvesting ability^7,22,23^. Knight et al.^24^ demonstrated for the first time that an array of rod-shaped Au nanoantennas allows NIR light to trigger a current response on a silicon-based photodiode, even though the wavelength (1250–1600 nm) is well below the semiconductor band gap. The NIR-promoted e^−^/h^+^ pairs arise exclusively from non-radiative damping of surface plasmons on Au. More importantly, they become accessible to a Si diode with the aid of a Schottky junction at the metal-to-semiconductor interface, in which the energy barrier is much smaller for those energetic or hot electrons than the band gap of Si. The significance of these results lies in corroborating the propensity of NIR light to induce useful energetic e^−^/h^+^ pairs in photoactive devices.

Recently, those hot charge carriers prompted by NIR-excited plasmons were reported not only to generate electricity^24–27^ but also to support chemical fuel generation^20,21,28^, thereby addressing a paradigm in photocatalysis. Hence, Mubeen et al.^28^ reported on the evidence of hydrogen (H_2_) gas evolution on a photoexcited dense Au nanorod (NR) array that was sprinkled atop with a bifunctional TiO_2_ semiconductor and laterally with a cobalt-based oxygen-evolution catalyst (Co-OEC) in the presence of a sacrificial agent. Furthermore, Zheng et al.^21^ introduced an alternative of strewing the tips of Au NRs merely with Pt in lieu of TiO_2_ and Co-OEC. The dual functionality of Pt, on the one hand, includes serving as an electron sink to prevent hot electrons to drift back to the Au NRs. This is also the asset and function of a Schottky junction. Moreover, the catalytic effect of Pt allows H_2_ generation in the presence of a photohole scavenger. These results validate the persistence of energetic electrons in producing H_2_ upon photoexcitation of Au NR with visible-NIR light radiation. However, the direct exposure of the plasmonic metals to the electrolyte can result in undesirable corrosion and/or dissolution of the Au NR during reaction^22,23,29^. As an alternative, we here suggest a metal-semiconductor composite structure with the plasmonic nanoantennas embedded in the semiconductor, emerging as a breakthrough to retrieve the aforementioned depleted photoactivity. Even more important, the concomitant 3D heterojunction of such metal-semiconductor composite can also support hot-electron injection due to the substantially increased metal-to-semiconductor interface^27,30^.

In this contribution, we implanted Au@Nb core-shell nanoparticles (CS-NP) into the cavity of tubular protonated metaniobate (H_*x*_K_1−*x*_NbO_3_) nanoscrolls (NSs). In particular, this infiltration emulates the design of a natural plant, namely, peapods. Thus, Au@Nb CS-NPs are unidirectionally deposited yet mutually isolated with a break of few nanometers in between. In addition to the passivation against photocorrosion and the enhanced Schottky junction area, the biomimicry of this peapod design endow the Au@Nb@H_*x*_K_1−*x*_NbO_3_ nanopeapod (NPP) photocatalyst with an exceptional broadband photoabsorption ability that eminently matches with the solar spectrum. In particular, the outmost H_*x*_K_1−*x*_NbO_3_ semiconductor NS (the pod) is responsible for UV light absorption, whereas the inner Au@Nb CS-NPs (the peas) account for visible light absorption via surface plasmon resonance (SPR). Most significantly, this nanopeapod design gives rise to a strong near-field interantenna coupling that additionally creates the excellent photoresponse of Au@Nb@H_*x*_K_1−*x*_NbO_3_ NPPs to NIR light. To the best of our knowledge, this peculiar NPP design is the first alternative pattern—in addition to the geometry and size—to effectively expand the resonant plasmon wavelength of Au nanoantennas over the full solar spectrum. The resulting effective NIR-induced dye decolorization and water cleavage in the absence of an irreversible whole scavenger substantiate the relevance and potential of the peapod-structured photocatalyst.

## Results

### Synthesis and characterization of Au@Nb@H_*x*_K_1−*x*_NbO_3_ nanopeapods

The peapod design is foremost characterized by the tubular morphology of the semiconductor. Moreover, the passivation of the encapsulated plasmonic metal is a most relevant aspect to avoid its dissolution due to photocorrosion processes. To this regard, oxide-based materials known for high stability—particularly, TiO_2_, α-Fe_2_O_3_ or niobates—are promising semiconductors that were already shown to form tubular nanostructures^31–33^. Hereof, H_*x*_K_1−*x*_NbO_3_ is favored due to its delicate band-edge position straddling most redox reactions of interest, and due to the *d*-orbital nature of its conduction band rendering abundant density of states (DOSs) for a rapid electron uptake^34,35^.

**Fig. 1:**
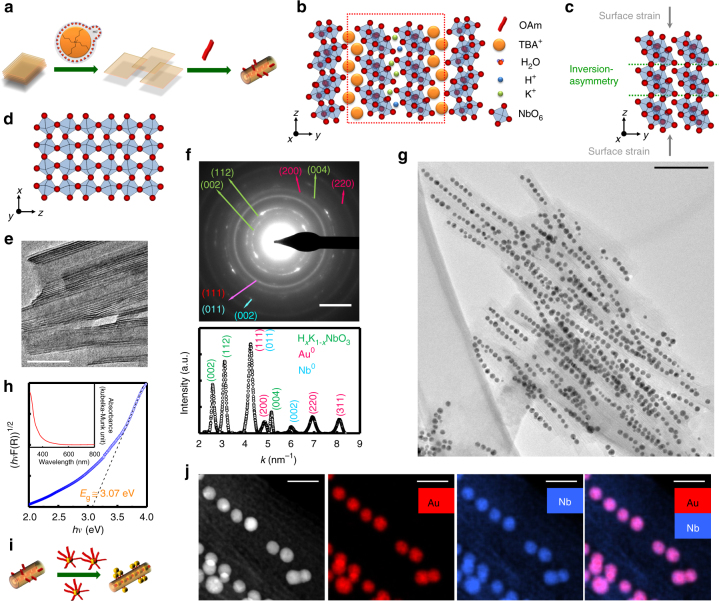
Stepwise formation and characterization of Au@Nb@H_*x*_K_1−*x*_NbO_3_ NPPs. **a** Schematic and **b**, **c** crystallographic illustrations of the morphological evolution of templated H_*x*_K_4−*x*_Nb_6_O_17_ crystallites to lamellar and tubular colloids. **d** Crystal structure of cubic HNbO_3_ perovskite. **e** TEM image (scale bar: 70 nm) of tubular H_*x*_K_1−*x*_NbO_3_ NSs, in which the hollow cavity functions as the host to breed the Au@Nb CS-NPs. **f** SAED pattern (top, scale bar: 3 nm), corresponding azimuthally averaged SEAD pattern (bottom) and **g** TEM image (scale bar: 100 nm) of the Au@Nb@H_*x*_K_1−*x*_NbO_3_ NPPs. **h** Absorption spectrum (inset) and derived Tauc plot of the H_*x*_K_1−*x*_NbO_3_ NSs. **i** Schematic highlights of the biomimicry of the Au@Nb@H_*x*_K_1−*x*_NbO_3_ NPPs, wherein the Au@Nb CS-NPs as the “peas” are unidirectionally loaded into the tubular cavity of nanoscale H_*x*_K_1−*x*_NbO_3_ as the “pods”. **j** HAADF-STEM image and EDXS elemental maps (scale bars: 20 nm) of the Au (red), Nb (blue), and combined Au/Nb distribution prove the structure of the Au@Nb@H_*x*_K_1−*x*_NbO_3_ NPPs, in which the Au@Nb CS-NPs (lilac) are unidirectionally located inside the H_*x*_K_1−*x*_NbO_3_ NSs (blue). Abbreviations used: OAm, oleylamine; TBA^+^, tetrabutylammonium cation; NS, nanoscroll; CS-NPs, core-shell nanoparticles; NPP, nanopeapod

Preparing H_*x*_K_1__−__*x*_NbO_3_ directly from either HNbO_3_ or KNbO_3_ in an anisotropic tubular shape, however, is not straightforward as both have a cubic structure^36,37^. Such cubic symmetry typically leads to the formation of an isotropic crystallite growth most often resulting in cubes or octahedra^6,36^. To fabricate tubular H_*x*_K_1−*x*_NbO_3_ either by protonating of KNbO_3_ or by exchange of protons (H^+^) in HNbO_3_ by potassium (K^+^) ions seems highly unlikely. Here we have addressed this issue by using the orthorhombic potassium hexaniobate (K_4_Nb_6_O_17_) as the starting material, which already has a lamellar structure and which is thus ideal as a template^38–40^. Hence, the synthesis started with the K_4_Nb_6_O_17_ template that was subsequently subjected to a cascade of reactions mainly including an interlamellar ion exchange (i.e., H_*x*_K_4−*x*_Nb_6_O_17_) to degenerate its structural integrity followed by an exfoliation process of individual lamella by bulky tetrabutylammonium (TBA^+^) ions (Fig. 1a–c). The resulting isolated molecular sheets then undergo automatic curling due to an intrinsic lattice asymmetry inside each lamella and finally forms structured tubular NSs^38^. Hereof, the last two steps using a strongly hydrated tetrabutylammonium hydroxide and an organic media are the key to promote the desired phase transformation, in which the dissociation of coordinated water molecules from TBA^+^ was retarded that otherwise promote the exfoliated H_*x*_K_4−*x*_Nb_6_O_17_ lamella to undergo hydrolysis at high temperature according to Equation (1)^36,40^.

1 $${\rm H}_x{\rm K}_{4 - x}{\rm Nb}_6{\rm O}_{17} + {\rm H}_2{\rm O} \to 6{\rm H}_x{\rm K}_{1 - x}{\rm NbO}_3.$$

It is worth mentioning that the H_*x*_K_4−*x*_Nb_6_O_17_ sheets consist of a central layer of [NbO_6_] octahedra shearing corners in the [100] direction and alternating edges and corners in the [001] direction (Fig. 1c)^36,37^. Such corner-shearing [NbO_6_] octahedra are also an inherent structural feature of the H_*x*_K_1−*x*_NbO_3_ perovskite (Fig. 1d) serving as a backbone and facilitating the phase transformation. Such templated crystallization can be regarded as topotactic reaction, in which the template is subjected to a phase transition without sacrificing its morphology (Fig. 1e). Selected area electron diffraction (SAED) patterns (Fig. 1f, top) and azimuthally averaged SAED patterns (Fig. 1f, bottom) of an Au@Nb@H_*x*_K_1−*x*_NbO_3_ NPP ensemble show (002), (112), and (004) reflections belonging to the H_*x*_K_1−*x*_NbO_3_ perovskite (space group *Im*-*3*, *a* = 7.65 Å). In addition, reflections of pure Au (face-centered-cubic structure, space group *Fm-3m*, *a* = 4.08 Å) and Nb (body-centered cubic structure, space group *Im-3m*, *a* = 3.32 Å) are visible. Geometric features of the finally formed H_*x*_K_1−*x*_NbO_3_ NSs are delineated by transmission electron microscopy (TEM) with an orifice diameter of about *d* = 20 nm and an outer scroll diameter in a range of *D* = 35–70 nm (Fig. 1e), while the length of the NSs lies between 0.1 and 1.0 μm (Fig. 1g). The photoabsorption, which is another crucial property with regard to photocatalysis application, was characterized through diffuse reflectance spectroscopy (DRS). Hence, a featured absorption edge at 390 nm (Fig. 1h, inset) results in a band gap of 3.07 eV (Fig. 1h) for the H_*x*_K_1−*x*_NbO_3_ NSs, which is in good agreement with the values reported in the literature^41,42^. A slight decrement of the band gap is presumably related to weakened Nb–O–Nb bonds originating from the curling of the niobate layers to form the NSs^43^.

To date, there is a variety of technologies for fabricating 1D Au peapodded nanostructures, including vapor–liquid–solid (VLS) growth and atomic layer deposition (ALD)^44–46^. However, the Rayleigh perturbation at the tempering step in those approaches generally generates NPPs with an interparticle spacing (*d*_gap_) of nearly 100 nm which fairly exceeds the diameter of the Au particles (*d*_Au_) and which leads to a ratio of interdistance over diameter (*d*_gap_/*d*_Au_) >1. Such giant spacing hinders the surface plasmon polaritons (SPPs) on photoexcited Au NPs from electric near-field coupling^47–50^. As a consequence, a waning photoresponse at excitation wavelengths remote from the characteristic SPR was reported^44–46^. In order to retrieve the fading plasmon coupling, this article tackles this challenge by taking a dissolved Au precursor in place of a solid Au catalyst to exclude the necessity of any thermal treatment^51^. Moreover, such solution-processed Au precursors can readily be capillarily engulfed into the cavity of the preformed H_*x*_K_1−*x*_NbO_3_ NSs. Afterwards, the employment of a reducing agent (i.e., oleyamine) allows the Au NPs to seed directly inside the semiconductor NSs (Fig. 1i). Figure 1g, j summarize TEM, high-angle annular dark-field scanning TEM (HAADF-STEM) and energy dispersive X-ray spectroscopy (EDXS) results that clearly depict the morphology and the Au and Nb distribution of the as-prepared NPPs. The TEM images (Fig. 1g, j) highlight the uniformity and filling efficacy while the unidirectionally aligned bright spots in HAADF-STEM images and the distinct elemental contrast in EDXS mappings (Fig. 1j) point to the arrangement of the Au NPs and the niobate NSs in the Au@H_*x*_K_1−*x*_NbO_3_ NPPs, respectively.

### Characterization of the Au@Nb core-shell nanoparticles

**Fig. 2:**
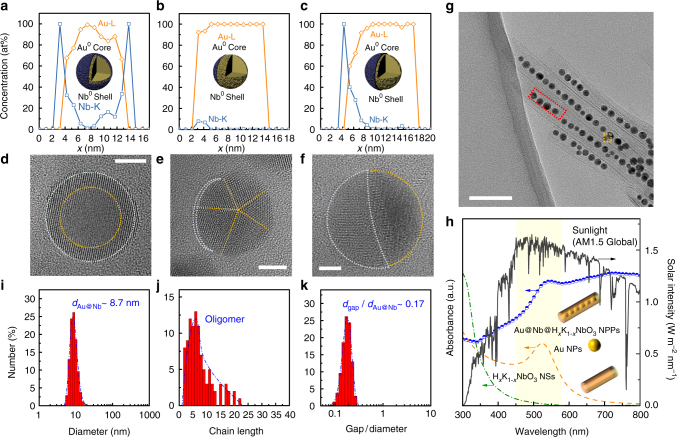
CS-NP configuration and configuration-dependent surface plasmon resonances of the Au@Nb@H_*x*_K_1−*x*_NbO_3_ NPPs. **a**–**c** EDXS line profiles (element contributions from the H_*x*_K_1−*x*_NbO_3_ NSs were subtracted) and **d**–**f** corresponding HRTEM images reveal the CS-NP configuration of the Au@Nb@H_*x*_K_1−*x*_NbO_3_ NPPs. Orange and white dashed lines in the HRTEM images delineate core and shell boundaries (lines are guide to the eye) of single-crystalline Au@Nb CS-NPs with a continuous Nb shell (**a**, **d**) (scale bar: 4 nm); multiply twinned Au NPs with thin incomplete Nb shells (**b**, **e**) (scale bar: 3 nm); Au@Nb CS-NPs with a partial Nb shell (**c**, **f**) (scale bar: 2 nm). **g** TEM overview image (scale bar: 50 nm) of the Au@Nb@H_*x*_K_1−*x*_NbO_3_ NPPs showing the Au@Nb CS-NPs located within (red frame) and on (orange frame) the H_*x*_K_1−*x*_NbO_3_ NSs. **h** Absorption spectrum of the Au@Nb@H_*x*_K_1−*x*_NbO_3_ NPPs (half-filled circles). AM 1.5G solar spectrum (solid line), naked Au NPs (dashed line), and hollow H_*x*_K_1−*x*_NbO_3_ NSs counterparts (dash-dotted line) are plotted also for comparison. **i**–**k** Pattern features of the Au@Nb CS-NPs with **i** particle size, **j** length of continuous CS-NP chains (in number of CS-NPs per chain) and **k** interparticle distance in units of Au@Nb particle size, respectively. Experimental data determined from different TEM images and fits of these data (blue dashed lines). Abbreviations used: *d*_Au@Nb_, diameter of bimetallic Au@Nb nanoparticle; *d*_gap_, interparticle distance between adjacent Au@Nb nanoparticles of the Au@Nb@H_*x*_K_1−*x*_NbO_3_ NPP; NSs, nanoscrolls; NPs, nanoparticles; NPPs, nanopeapods

The high yield of the Au@H_*x*_K_1−*x*_NbO_3_ NPPs distinctly indicates a strong contrast between Au nucleation rates outside and inside the niobate NSs. This discrepancy originates from the presence of oleyamine (OAm) at the earliest scroll formation stage (Fig. 1a)^40,51^. Hence, OAm can saturate the cavity of the NSs and accelerates the growth rate of inner Au nanopeas. Surprisingly, EDXS (Fig. 1j) of those inner Au nanopeas reveals an exceptional effect of this preliminary treatment. Namely, OAm intercepts the niobium (Nb^5+^) leakage from H_*x*_K_4−*x*_Nb_6_O_17_ during the phase transition to the final H_*x*_K_1−*x*_NbO_3_ that contains a lower stoichiometric content of Nb^36^. The excess Nb^5+^ in turn is deposited as Nb^0^ atop the nucleated Au NPs (element maps in Fig. 1j; concentration profiles across single NPs in Fig. 2a–c) via underpotential deposition (UPD), resulting in the exceptional Au@Nb@H_*x*_K_1−*x*_NbO_3_ multi-core-shell peapod structure (Supplementary Figs 1–3, see Supplementary Methods and Supplementary Note 1 for details). Au@Nb CS-NPs with different Nb distributions are observed by high-resolution (HR) TEM (Fig. 2d–f) and the corresponding Nb-line profiles across single CS-NPs (Fig. 2a–c). Figure 2d shows a CS-NP with single-crystalline structure and a continuous Nb shell with a thickness of 1–2 nm (Fig. 2a). We note that H_*x*_K_1−*x*_NbO_3_ NSs cannot be seen in the HRTEM images due to their low contrast compared to the CS-NP contrast, but they can be well recognized in TEM overview images (Fig. 2g).

CS-NPs within H_*x*_K_1−*x*_NbO_3_ NSs (red frame in Fig. 2g) are typically covered by a continuous Nb shell as demonstrated by Fig. 2a, d. The formation of a pure Nb shell (instead of Nb oxide) is further supported by its structure, which corresponds to bulk cubic Nb (Supplementary Fig. 4). Inhomogeneous Nb distributions are found for CS-NPs at the outer surface of the H_*x*_K_1−*x*_NbO_3_ NSs. For example, the CS-NP in Fig. 2e is only partially covered by a Nb shell (Fig. 2b). The Au core of this NP exhibits a multiply twinned structure with a decahedral shape along its fivefold symmetry axis (Supplementary Fig. 5). The CS-NP in Fig. 2c, f forms a Nb bridge at the interface toward the niobate NSs. The position of this CS-NP is marked by an orange frame in Fig. 2g. The Bragg reflections of the CS-NPs are also present in the SAED pattern (Fig. 1f), where Au (200), (220), and (311) diffraction rings with a uniform intensity support the conclusions from the HRTEM analysis. The intensity of the Nb reflections is in general weak due to the small Nb amount contained in the CS-NPs but the Nb (002) reflection can be clearly recognized in the azimuthally averaged SAED pattern (Fig. 1f, bottom). These analyses show exemplary features of bimetallic CS-NPs prepared by an UPD process, which refers to the deposition of a metal film with the thickness up to few atomic layers on a foreign metal substrate at a potential much more positive than that for the deposition on the same metal surface^52^. Accordingly, UPD is the most likely mechanism for the formation of metallic Nb (standard reduction potential –0.64 V vs. SHE) by OAm in the presence of Au NPs.

### Broadband light harvesting ability

One of the important merits of the unique Au@Nb CS-NP formation is that the Nb shell can superimpose its characteristic SPR absorption upon that of the Au core to modulate the optical properties of the Au@Nb CS-NPs^53^. As a consequence, the Au@Nb@H_*x*_K_1−*x*_NbO_3_ NPPs produce an absorption spectrum (Fig. 2h), exhibiting a somewhat flatter nature at wavelengths beyond 400 nm (featured SPR absorption of Nb) yet below 530 nm (featured SPR wavelength of Au)^22,23,54^. Intriguingly, a similar scenario is also observed at wavelengths beyond the SPR frequencies of both the Au core and the Nb shell, namely, an intensive and continuous bathochromic spectral feature emerges in the red-NIR region (Fig. 2h). Evidently, this does not originate from the Au@Nb CS-NPs. One should therefore take another key component in Au@Nb@H_*x*_K_1−*x*_NbO_3_ NPPs into account, viz. is the niobate NSs. Bringing the metallic nanoantennas into contact with a dielectric NS, unlike the metal–metal interaction, generally leads to a redshift of the SPR^22,23,53^. This is illustrated by the SPR absorption of the Au@Nb@H_*x*_K_1−*x*_NbO_3_ NPPs in comparison to the uncovered Au core (yellowish tinted region in Fig. 2h). The absorption band in the red-NIR region; however, cannot yet be reasonably explained.

Taken together, we could neither call the individual ingredients nor an overall combination in a NPP to account for its peculiar red-NIR absorption. In other words, only the biomimicry of the peapod design, and especially, its specific configurational traits, that differ significantly from known peapod structures, are responsible for this absorption band (Fig. 2i–k)^44–46^. Thus, the diameter of the Au@Nb CS-NPs is highly monodisperse with an average of 9 nm (Fig. 2i), and the CS-NPs are intensively accumulated ranging from dimers to most often observed heptamers (Fig. 2j) that show uniform interparticulate distances of about 2 nm (Fig. 2k). Even more important, this interparticular distance is small in relation to the Au@Nb CS-NPs size and well below the theoretically suggested threshold to induce a desirable electric near-field coupling^47^. Such near-field coupling effect resembles the longitudinal SPR (LSPR) mode of an anisotropic Au nanoantenna and promotes an additional absorption band at wavelengths longer than the intrinsic transverse-mode SPR (TSPR) frequency (530 nm) of the Au NPs^47–50^. The uniaxially aligned Au@Nb CS-NPs result in a field enhancement particularly along the chain and lead to a LSPR-analogous absorption overwhelming nearly the entire red-NIR regime up to 800 nm.

### Photocatalytic dye decomposition

**Fig. 3:**
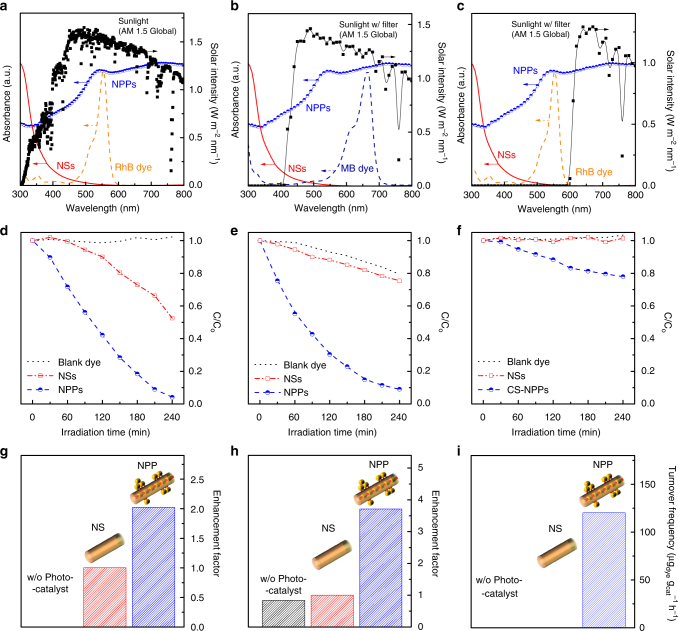
Broadband photocatalytic activity of the Au@Nb@H_*x*_K_1−*x*_NbO_3_ NPPs. **a**–**c** Spectra of simulated sunlight (**a**) further equipped with either an UV cutoff filter (**b**) or a colored glass filter (**c**) that work as irradiation sources for photocatalytic dye degradation. Absorption spectra of the employed H_*x*_K_1−*x*_NbO_3_ NSs (solid line) and the Au@Nb@H_*x*_K_1−*x*_NbO_3_ NPPs (half-filled circles) as photocatalysts; methylene blue (MB) and rhodamine B (RhB) as probing species (dashed line) are plotted alongside for reference. **d**–**f** Photocatalytic decoloring of RhB (**d**, **f**), and MB (**e**) in the absence (dotted line) or in the presence of either niobate NSs (empty squares) or Au@Nb@H_*x*_K_1−*x*_NbO_3_ NPPs (half-filled circles). **g**–**i** Comparison of the wavelength-dependent photoactivity between H_*x*_K_1−*x*_NbO_3_ NSs and Au@Nb@H_*x*_K_1−*x*_NbO_3_ NPPs under illumination with full solar light (**g**), visible-NIR integral light (**h**), and red-NIR integral light (**i**), respectively. Abbreviations used: MB, methylene blue; RhB, Rhodamine B; NSs, nanoscrolls; NPPs, nanopeapods; NIR, near-infrared

So far, we have specified the configuration-dependent SPR effect of the Au@Nb@H_*x*_K_1−*x*_NbO_3_ NPPs endowed with a broadband light harvesting ability. Next, photocatalytic dye decomposition was carried out to substantiate the effect of comprehensive photon accumulation to enhance the useful charge carrier concentration for chemical reactions (Fig. 3). The experiments were carefully designed by using a UV cutoff filter and colored glass filters (*λ* > 610 nm) (Fig. 3a–c) to distinguish the plasmon-initiated carriers from those originating from the semiconductor. Moreover, methylene blue (MB) and Rhodamine B (RhB) were employed as the probing molecules to avoid the photosensitized dye perturbing the evaluation (see Methods section for details)^34,55,56^.

In all cases, the presence of the Au@Nb CS-NPs substantially accelerates the H_*x*_K_1−*x*_NbO_3_ NS semiconductor to bleach the dye solutions (Fig. 3d–f). The highest decoloring efficiency is observed in the case of the Au@Nb@H_*x*_K_1−*x*_NbO_3_ NPPs under full solar illumination (Fig. 3d). Evidently, the charge carriers involved in the chemical conversion only partly arise from the niobate NSs upon UV excitation. Moreover, the Au@Nb CS-NPs offer extra carriers upon SPR that is responsible for the acceleration (Fig. 3g–i). Taken together, the plasmonic nanoantennas boost the carrier quantity in either populating the energetic electrons originally residing on the metal to the adjacent semiconductor (direct electron transfer, DET) or in resonantly transferring its plasmonic energy to generate more e^−^/h^+^ pairs on the semiconductor (resonant energy transfer, RET)^22,23,57,58^. Clearly, the enhancement in carrier concentration on the Au@Nb@H_*x*_K_1−*x*_NbO_3_ NPPs arises mostly from a DET in lieu of a RET process in terms of a minute spectral overlap between the Au NPs and the H_*x*_K_1−*x*_NbO_3_ NSs (Fig. 2h). In a DET process, electrons of a plasmonic metal receive energy from an interacting photon followed by traversing a metal-semiconductor interface—the Schottky interface to migrate into the CB of the semiconductor. On this basis, the internal quantum transmission probability (*η*_*i*_) for electron injection can be formulated by a modified Fowler equation^59,60^,

2 $$\eta _i \propto \frac{{(h\nu - q\phi _{\mathrm{b}})^2}}{{hv}},$$

where *hν* is the incident photon energy, and where *qΦ*_b_ is the barrier height of the Schottky junction. The barrier height is established by electronic alignment between the Fermi level of the plasmonic metal and the flat-band potential (*V*_fb_) of the coupled semiconductor. Herein, the work function of the Au@Nb CS-NPs is located in a spectrum with Au (~5.1 eV) and Nb (~4.3 eV) standing at the ends^61^. Given the insignificant fraction of a thin Nb shell in the Au@Nb CS-NPs (Fig. 2), the work function is primarily dictated by Au with a value close to 5.1 eV. *V*_fb_ of the H_*x*_K_1−*x*_NbO_3_ semiconductor was estimated by the equation reported by Scaife et al.^62^ for oxides that do not contain metal cations with partially filled *d*-orbital as follows,

3 $$V_{\mathrm{fb}}\left( {\mathrm {vs}.}\,{\mathrm {NHE}} \right) = 2.94 - E_{\mathrm {g}},$$

where *E*_g_ is the bandgap of the semiconductor. Consequently, a *V*_fb_ of –0.13 V (vs. NHE) for the H_*x*_K_1−*x*_NbO_3_ NSs and a barrier height of ~0.7 eV for the Schottky junction between the Au@Nb CS-NPs and the H_*x*_K_1−*x*_NbO_3_ NSs were derived. Surprisingly, this energy barrier is nearly one quadrant of the bandgap of the H_*x*_K_1−*x*_NbO_3_ semiconductor, which is the key to allow photons that are invisible to the niobate, to produce active carriers to be used in chemical reactions. More significantly, the strong peapod-specific T- and L-SPR phenomena (Fig. 2h) permit visible- and NIR-photons to efficiently lend electrons on Au@Nb CS-NPs sufficient energy to enter the H_*x*_K_1−*x*_NbO_3_ NSs and to contribute to the dye degradation^63^. This prima facie evidence is given by the steady and efficient photobleaching courses (Fig. 3e, f).

### Photoelectrochemical water splitting

In addition to the fundamental dye degradation study, the Au@Nb@H_*x*_K_1−*x*_NbO_3_ NPPs were examined in view of the photoelectrochemical (PEC) water splitting to corroborate the ability of the SPR-initiated photoinduced carriers in transferring solar energy into useful chemical fuels. In such artificial photosynthesis process (i.e., 2H_2_O → 2H_2_ + O_2_), highly valuable H_2_ molecules are produced under sunlight^3^, which highlights the merit of employing this reaction for evaluation. The evaluation of the respective photon energy storage efficiency is deduced from the overall reaction rate, which was mostly expressed by the photoinduced current flowing from the Au@Nb@H_*x*_K_1−*x*_NbO_3_ photoelectrode (Methods section). It is to be noted that an argon-purged aqueous solution containing 0.5 M Na_2_SO_4_ was used as the electrolyte and no sacrificial agent was present.

**Fig. 4:**
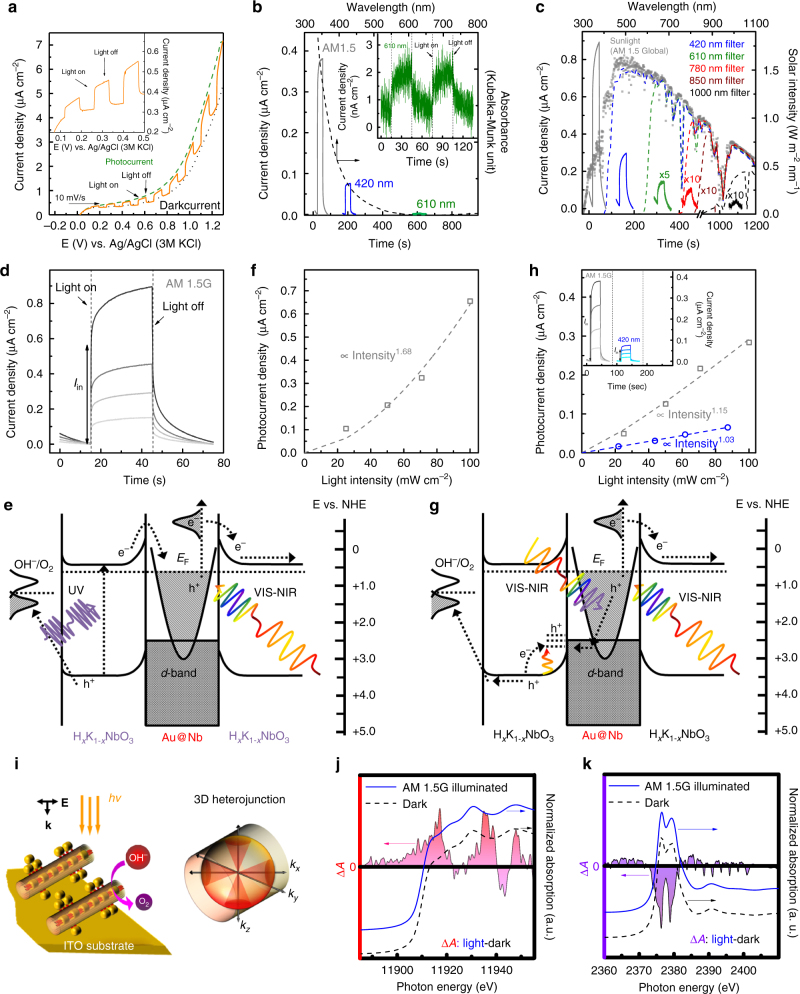
Photoelectrochemical water splitting with the Au@Nb@H_*x*_K_1−*x*_NbO_3_ NPP photoelectrode. **a** Current-potential characteristics of the Au@Nb@H_*x*_K_1−*x*_NbO_3_ photoelectrode in 0.5 M Na_2_SO_4_ solution (pH 6.8) under chopped (orange line) and continuous (green dashed line) AM 1.5G simulated sunlight illumination. The dark current is plotted alongside (dark dotted line). **b**, **c** Wavelength-dependent photocurrent-time plots (baseline subtracted) of a H_*x*_K_1−*x*_NbO_3_ NS (**b**) and **a** Au@Nb@H_*x*_K_1−*x*_NbO_3_ NPP photoelectrodes (**c**) under irradiation of AM 1.5G simulated sunlight (gray line) equipped with different long-pass filters (colored solid lines) in the presence of an anodic bias of 1 V (vs. Ag/AgCl). Absorption spectra of the H_*x*_K_1−*x*_NbO_3_ NSs (black dashed line in **b**) and AM 1.5G simulated sunlight (filled squares in **c**) illumination after passing a UV cutoff filter (blue dashed line in **c**) or different colored glass filters (green, red, brown, and black dashed line in **c**) are plotted alongside for comparison. **b** Photocurrent-time plot (baseline subtracted) of the H_*x*_K_1−*x*_NbO_3_ NS photoelectrode under integral red-NIR illumination in the presence of an anodic bias of 1 V (vs. Ag/AgCl). **d** Fluence-dependent photocurrent-time plots (baseline subtracted) of the Au@Nb@H_*x*_K_1−*x*_NbO_3_ NPP photoelectrode. **e** Postulated charge transfer on the Au@Nb@H_*x*_K_1−*x*_NbO_3_ NPP heterojunction starts with the photoabsorption process. **f** *I*_in_ as a function of simulated sunlight intensity of the Au@Nb@H_*x*_K_1−*x*_NbO_3_ NPP photoelectrode. **g** Postulated charge transfer on the Au@Nb@H_*x*_K_1−*x*_NbO_3_ NPP heterojunction via a two sequential one-photon absorption process. **h** *I*_in_ as a function of the intensity of either simulated sunlight (gray line) or integral VIS-NIR light (blue line) for the H_*x*_K_1−*x*_NbO_3_ NS photoelectrode. **h** Fluence-dependent photocurrent-time plots (baseline subtracted) of the H_*x*_K_1−*x*_NbO_3_ NS photoelectrode. **i** Schematic illustration of the 3D metal-semiconductor heterojunction of the Au@Nb@H_*x*_K_1−*x*_NbO_3_ NPP photoelectrode. **j**, **k** Normalized Au (**j**) and Nb (**k**) *L*_*3*_-edge XAS spectra of the Au@Nb@H_*x*_K_1−*x*_NbO_3_ NPPs collected in the presence and absence of AM 1.5G simulated sunlight. The difference in absorbance (∆A) between the aforementioned two conditions is plotted alongside. Abbreviations used: NS, nanoscroll; NPP, nanopeapod; VIS, visible, NIR, near-infrared, *I*_in_, initial photocurrent shoot at the light-on instant; *E*_F_, Fermi level; **E**, electric field, **k**, wavenumber; ITO, tin-doped indium oxide

The specific photocurrent-potential response of the Au@Nb@H_*x*_K_1−*x*_NbO_3_ photoelectrode was first characterized under chopped full solar illumination at a fluence of 100 mW cm^−2^ (Fig. 4a). The anodic photocurrent was first perceived at about 0.1 V (Fig. 4a) and raises steadily to 0.9 μA cm^−2^ at 1 V (i.e., potentials on the Ag/AgCl scale). The enhancement upon anodic polarization stems from the reinforced band bending within the space-charge layer of the outmost H_*x*_K_1−*x*_NbO_3_ NSs which effectively rectifies the carrier transport (Supplementary Fig. 6a, b, see Supplementary Note 2 for details)^64^. This anodically biased photocurrent doubles the performance of the H_*x*_K_1−*x*_NbO_3_ photoelectrode under the same conditions (Fig. 4b, c). Even more important, the photocurrent enhancement multiplies significantly upon modulating the irradiation wavelength. The enhancement factor calculated as the photocurrent of the Au@Nb@H_*x*_K_1−*x*_NbO_3_ NPP photoelectrode divided by that of the H_*x*_K_1−*x*_NbO_3_ NS photoelectrode is threefold below the integral of VIS-NIR light illumination and reaches one order of magnitude below the broadband red-NIR light illumination. Most surprisingly, the maximum improvement is achieved under NIR light irradiation alone, even though its photon energy (<1.8 eV) is far below that of visible (2–3 eV) and UV (>3 eV) photons. Moreover, the characteristic photocurrent-time response of the Au@Nb@H_*x*_K_1−*x*_NbO_3_ photoelectrode was monitored (at 1 V vs. Ag/AgCl), in which the photocurrent shows an intriguing transient behavior (Fig. 4d). In the light-on period, the photocurrent demonstrates an initial shoot (*I*_in_) followed by a steady raise mimicking the rectangular signal that is expected in an ideal case^64^. This variation implies a non-equilibrium between the carrier generation rate under light casting and its consumption rate upon reacting with water molecules. Several probable mechanisms may account for that and are inspected in detail below.

Considering the thermodynamically (△_*r*_*G*^0^ = + 472 KJ mol^−1^) and kinetically (four-electron oxidation: 2H_2_O → 2O_2_ + 4 H^+^ + 4e^−^) unfavorable cleavage of water, the poor water oxidation on the Au@Nb@H_*x*_K_1−*x*_NbO_3_ NPP photoelectrode can influence the PEC performance. For this reason, 1 M Na(COOH) was added to the electrolyte to modify the overall reaction mechanism. The oxidation of formate is comparably easy (one-electron oxidation: [HCOO]^−^ → CO_2_ + H^+^ + e^−^)^65^. As a consequence, the photocurrent is nearly doubled in the presence of Na(COOH) (Supplementary Fig. 7a–c, see Supplementary Note 2 for details). Nonetheless, the independence of the photocurrent transient implies that the reaction kinetics of the chemical reaction is not the major contribution. In other words, the charge-carrier generation kinetics is responsible for the phenomenon. This finding encourages us to postulate the following mechanism for the different photocurrent response (Fig. 4d). Under broadband sunlight illumination, charge carriers occur simultaneously on the H_*x*_K_1−*x*_NbO_3_ NS semiconductor and the Au@Nb CS-NP nanoantenna upon UV and integral VIS-NIR light excitation (Fig. 4e). The SPR-mediated energetic electrons on the Au@Nb CS-NPs become the charge carriers on the H_*x*_K_1−*x*_NbO_3_ NS semiconductor via the DET process^57,58^. An external bias then dictates these charges, including the metal-injected and the semiconductor-originating electrons—to move to the Pt electrode for water reduction to H_2_^3^. Meanwhile, the photogenerated hole on the H_*x*_K_1−*x*_NbO_3_ NS semiconductor is scavenged by the surrounding aqueous medium to generate O_2_ on the Au@Nb@H_*x*_K_1−*x*_NbO_3_ NPP photoanode (Supplementary Figs. 8 and 9, see Supplementary Note 2 for details). Finally, a second UV photon produces the next e^−^/h^+^ pair on the H_*x*_K_1−*x*_NbO_3_ NS semiconductor followed by an electron transfer to a Au@Nb CS-NP to compensate the accumulated hole and to complete the overall process^66,67^.

The above process suggests a linear dependency of the photocurrent on the intensity of incident simulated sunlight (see Supplementary Note 3 for details), which, however, is in significant contrast to the superlinear behavior shown in Fig. 4f. The quasi-quadratic correlation indicates a nonlinear effect in the process of the photohole compensation. In brief, the electron vacancies on the Au@Nb CS-NPs are first subjected to an excitation process to a lower energy state in the *d*-band on the Au@Nb CS-NPs upon electron transition of the subsequently incident photon (Fig. 4g). This view is strongly supported by Jiang et al.^68^ who reported on a similar phenomenon regarding the photoluminescence of anisotropically coupled Au NPs. The overall process—designated as two sequential one-photon absorption in the literature—starts with a first VIS-/NIR-photon inducing a *sp* → *sp* intraband transition to create an electron vacancy below the Fermi level of certain plasmonic nanostructure, which is in analogy to the SPR-mediated hot-electron generation process on the Au@Nb CS-NPs in our study. The second photon then excites an electron from the *d*- to the *sp*-band via an interband transition to transfer the hole to the *d*-band. According to Jiang et al.^68^, the *d*-band hole recombines with the electron in the *sp*-band, giving rise to photoluminescence. In contrast, the photocurrent response in our study indicates that the hole is retrieved by the coupled H_*x*_K_1−*x*_NbO_3_ NS semiconductor, and thereafter it participates in water oxidation in preference to charge recombination.

In this hole recovery, energy states associated to H_*x*_K_1−*x*_NbO_3_ defects at the interface between the Au@Nb CS-NPs and the H_*x*_K_1−*x*_NbO_3_ NSs play an important role. The surface states originate mostly from the stepwise nano-texturization of the H_*x*_K_1−*x*_NbO_3_ NSs, as evidenced by the absorption spectra (i.e., the inefficient absorbance at wavelengths beyond the band gap) and the photocurrent-time plot (i.e., the photocurrent response under sub-bandgap integral VIS-NIR light irradiation). In general, these mid-gap electronic states are identified with a shallow energy position close to the valence band (VB) edge, which favors them to serve as a hole relay to facilitate the extraction^58,64^. Afterwards, a thermal transition brings the hole from this trapped state to the VB of the H_*x*_K_1−*x*_NbO_3_ NS semiconductor, where it drifts to the surface and reacts with water molecules to generate O_2_. This cascade-type charge delivery explains the superlinearity of the transient photocurrent response of the Au@Nb@H_*x*_K_1−*x*_NbO_3_ NPP photoelectrode on the light intensity. Noteworthy, the probability of the intraband transition predominates that of the interband transition in the very beginning of the light-on phase, because the electron density in the *sp* band is much higher than that of the photogenerated *sp* band vacancy. Afterwards, the quantitative surge in the *sp*-band hole in turn reinforces the probability of the interband transition (Supplementary Fig. 10a–f, see Supplementary Note 3 for details), which is responsible for the current rise following *I*_in_ in the photocurrent-time plot (Fig. 4d).

Despite of the photohole transfer subjected to the above described cascade mechanism, the photocurrent response is yet clearly visible under NIR light illumination (Fig. 4c). This implies a significantly mitigated recombination of antipodal charge carriers. In particular, such carrier loss in the volume of the H_*x*_K_1−*x*_NbO_3_ NSs is efficiently suppressed, as evidenced by the quasi-linear dependency demonstrated by the H_*x*_K_1−*x*_NbO_3_ photoelectrode without plasmonic Au@Nb nanoantennas (Fig. 4h). This can be attributed to the depletion region developed due to the applied bias that nearly overwhelmes the complete niobate NS (Supplementary Fig. 6a, b, see Supplementary Note 2 for details). Likewise, the carrier decay in the SPR excitation process is significantly quenched, which most likely relates to rapid spatial separation at the instant of the hot-electron generation. One should keep in mind that the isolation of the hot electron with an energy exceeding the Schottky-barrier height has to reach the metal-semiconductor interface to allow the DET process^59^. This propagation of a hot electron requests a momentum change (∆*k*) that is harvested from the wavevector (*k*-vector) of the incident photon with additional support of a phonon or an imperfection^69^. Such defect-assisted propagation suggests that the energetic electrons are preferentially formed at the margin of the sub-10-nm-sized Au@Nb CS-NPs due to the enormous imperfection area available at the boundary of the H_*x*_K_1−*x*_NbO_3_ NSs and the plasmonic CS-NPs. Herein, a considerable bridge area offered by the peapod-specific 3D metal-semiconductor interface greatly favors the H_*x*_K_1−*x*_NbO_3_ semiconductor to immediately collect those spatially localized hot electrons (Fig. 4i)^30,35^. Moreover, an energy dissipation due to electron collision is also circumvented and results in a high-DET efficiency. To this concern, X-ray absorption spectroscopy (XAS) at the Au and Nb *L*_*3*_-edge—describing the empty Au 5*d*/6*s-p* hybridized orbitals and the unoccupied Nb *4d* states being most responsible for the CB nature of the H_*x*_K_1−*x*_NbO_3_—was employed to study the performance. At first sight, a clearly increased absorbance is evidenced by Au *L*_*3*_-edge XAS after simultaneously irradiation with simulated sunlight, which indicates an additional electron transition from the Au 2*p* to the *s*-*p*-*d* hybridization orbitals by soft X-rays (Fig. 4j). This finding implies that additional electron vacancies are formed in this hybridized band. Concurrently, Nb *L*_*3*_-edge XAS shows a decreased absorbance that suggests reduced empty states in the Nb *4d* orbital being available to X-ray excited electrons from the Nb 2*p* state (Fig. 4k). Otherwise, the CB of H_*x*_K_1−*x*_NbO_3_ is occupied. Such inversed absorbance of the Au and the Nb *L*_*3*_-edge XAS reveals substantial evidence for the conversion of SPR-mediated hot electrons into the CB of H_*x*_K_1−*x*_NbO_3_ and validates their spatial isolation from the geminate photoholes^70^. Overall, the synergism between the rapid DET process and the cascade hole delivery is illustrated by the constant photocurrent flow at the incidence of modulated sunlight.

In summary, an alternative promising configuration of a plasmonic photocatalyst design is introduced emulating the growth pattern of a natural plant—a peapod. Spherical Au@Nb core-shell plasmonic nanoantennas with a nanometric break in between are unidirectionally deposited inside the cavity of a tubular H_*x*_K_1−*x*_NbO_3_ semiconductor. In particular, the biomimicry of this peapod design induces a strong interantenna electric near-field coupling that endows the Au@Nb@H_*x*_K_1−*x*_NbO_3_ nanopeapod photocatalyst with a NIR photon harvesting ability. More importantly, the collected NIR photons are exclusively transferred into energetic charge carriers via a direct electron transfer (DET) process to promote highly relevant chemical reactions, such as the degradation of organic dyes or water splitting (see Supplementary Note 4 for details).

For both organic dye degradation and water splitting, the photoactivity of the Au@Nb@H_*x*_K_1−*x*_NbO_3_ nanopeapod photocatalyst outperforms a photocatalyst without Au@Nb nanoantenna significantly. The enhancement stems mostly from the synergistic effect between (i) a rapid DET process due to the appreciable interfacial area provided by a peapod-specific 3D metal-semiconductor junction boosting hot-electron collection, and (ii) a cascade hole delivery supported by a sequential two-photon-assisted hole excitation process with rapid hole injection into the H_*x*_K_1−*x*_NbO_3_ semiconductor that is mediated by a mid-gap relay state. Taken together, the results suggest that the biomimetic pattern holds a great promise as a key design for widespread photoactive devices involving the use of NIR photons. This includes phototheranostic agents, optical sensors, plasmonic waveguides, broadband solar cells and so forth.

## Methods

### Fabrication of the H_*x*_K_1−*x*_NbO_3_ nanoscrolls

Templated K_4_Nb_6_O_17_ was prepared by a solid-state reaction, in which reagent-grade K_2_CO_3_ and Nb_2_O_5_ (99.99%) with a molar ratio of 1.1:1.5 were first ground together and heated in an alumina crucible at 900 °C for 1 h before continuing to 1050 °C for another 24 h. A slight excess of K_2_CO_3_ (10 mol-%) was employed to compensate for any material volatilized during heating. The cooling product was washed three times with distilled water and acetone respectively and dried in the oven overnight. The as-prepared K_4_Nb_6_O_17_ was next immersed in a warm (60 °C) and concentrated (3 mol/L) HCl solution for at least 4 days to obtain the proton-exchanged hexaniobates (H_*x*_K_4−*x*_Nb_6_O_17_). After the acid-treatment, the product was centrifuged and washed with distilled water and acetone, three times in each case, and dried overnight. Templated phase transformation from H_*x*_K_4−*x*_Nb_6_O_17_ to H_*x*_K_1−*x*_NbO_3_ and concurrent NS formation were carried out based on a solvothermal process reported by Adireddy et al.^40^ with some modifications. Experimental conditions were described below, the reaction solution was prepared by putting H_*x*_K_4−*x*_Nb_6_O_17_ (0.5 g), tetrabutylammonium hydroxide hydrate (TBAOH·30H_2_O, 0.75 g, 0.95 mmol) and oleylamine (OAm, 25 mL, ~75 mmol) together in 40 mL toluene. This mixture was magnetically stirred at ambient temperature for 1 h before transferring into a Teflon-lined stainless steel autoclave. The autoclave was then maintained at 220 °C (ramping rate = 2 °C/min) for 6 h and followed by naturally cooling. The resulting H_*x*_K_1−*x*_NbO_3_ NSs were collected by centrifugation, washed with ethanol several times and then dried overnight.

### Synthesis of the Au@Nb@H_*x*_K_1−*x*_NbO_3_ nanopeapods

In a standard synthesis, the preformed H_*x*_K_1−*x*_NbO_3_ NSs (20 mg), HAuCl_4_·4H_2_O (10 mg, ~0.025 mmol), oleic acid (OAc, 160 μL, 0.5 mmol), and OAm (165 μL, 0.5 mmol) were added to 3 mL hexane. The solution was then vigorously stirred and heated to nearly 60 °C for 24 h before cooling to room temperature. The color of the reacting mixture gradually turned from white to purple, suggesting the formation of Au@Nb CS-NPs inside H_*x*_K_1−*x*_NbO_3_ NSs. The obtained composites were purified by a repetitive dispersion/precipitation cycle with ethanol and finally dispersed in either deionized water or hexane for subsequent photo(electro)catalytic examinations.

### Analytical techniques

Samples for transmission electron microscopy (TEM) were prepared by casting one drop of hexane solution with Au@Nb@H_*x*_K_1−*x*_NbO_3_ NPPs onto a 300 mesh copper grid with carbon support film. The size, the morphology and the spatial distribution of Au@Nb CS-NPs and H_*x*_K_1−*x*_NbO_3_ NSs in Au@Nb@H_*x*_K_1−*x*_NbO_3_ NPPs were investigated by high-resolution (HR) TEM performed with an aberration-corrected FEI Titan^3^ 80–300 microscope operated at 300 kV. Moreover, a Philips CM200 FEG/ST microscope operated at 200 kV was utilized to perform selected area electron diffraction (SAED) for crystal structure and phase identification. SAED patterns of NPPs often show only few and rather diffuse reflections on Debye-Scherrer rings at large reciprocal lattice distances. A way to prevent the loss of valuable diffraction information is achieved by azimuthally averaging the intensity of the SAED pattern (for short radial scans), which is obtained by a 2*π* intensity integration of each spatial frequency ${k} = \left| {\mathbf k} \right|$ in the initial SAED pattern. Radial scans can be used to investigate the structure on NPs similar to XRD patterns. Lattice parameters and crystal structure of NPP material are determined from radial scans by using the peak positions obtained by fitting a Voigt function to each individual peak profile after subtraction a linearly interpolated background. Alternatively, HRTEM images were evaluated by calculating the two-dimensional Fourier transform, denoted as diffractogram, which yields information on the crystal structure (lattice parameters and crystal symmetry) of single nanoparticles. Crystal structure analyses was performed by comparing the experimental diffractograms and calculated diffraction patterns with Miller indices, where the latter were obtained by using the JEMS software. The absorption spectra of H_*x*_K_1−*x*_NbO_3_ NSs and Au@Nb@H_*x*_K_1−*x*_NbO_3_ NPPs were measured using a Varian Cary 100 Spectrometer and further analyzed by means of Kubelka-Munk transformation. For comparison, the ligand-stabilized Au NPs were additionally prepared in the same procedure as the fabrication of Au@Nb@H_*x*_K_1−*x*_NbO_3_ NPPs but devoid of H_*x*_K_1−*x*_NbO_3_ NSs. The as-obtained Au NPs were stored in hexane to collect an extinction spectrum.

### Photocatalytic dye decomposition measurements

The photocatalytic activities of Au@Nb@H_*x*_K_1−*x*_NbO_3_ NPPs and H_*x*_K_1−*x*_NbO_3_ NSs were evaluated based on the enhancement in degradation rate of Rhodamine B (RhB) and methylene blue (MB) at their presence. The reaction was carried out in a Pyrex reaction cell containing a suspension of the photocatalyst powder (50 mg) in 100 mL aqueous dye solution with continuous agitation using a magnetic stirrer. The employed dye concentration was 5.44 mg/L while a threefold diluted solution was used in evaluating the photoactivity under red-NIR light illumination. Before illumination, the suspensions were magnetically stirred in the dark for 1 h to ensure the establishment of an adsorption/desorption equilibrium of dyes on the photocatalyst surface. Afterwards, the reactions were conducted under irradiation of simulated AM 1.5 G solar light (100 mW/cm) using a LOT Class ABA solar simulator (type LS0805) as the light source. For the measurements under visible-NIR or red-NIR light, an additional UV cutoff (*λ* > 420 nm) or colored glass filter (*λ* > 610 nm) was used, respectively. The UV cutoff filter was employed to distinguish carriers produced by the UV-active H_*x*_K_1−*x*_NbO_3_ semiconductor from the plasmon-initiated carriers. Moreover, the colored glass filter was deployed to subdivide the SPR-initiated carrier formation into the visible-responsive transverse mode and the red-NIR-sensitive longitudinal mode, respectively. Since a charge transfer via the photosensitized dye may perturb reliable evaluation of the aspired SPR-boosted photon-to-carrier conversion efficiency in a dye degradation study, special attention needed to be paid to the probing molecules^55^. Herein, MB was chosen owing to the energetic mismatch between the lowest unoccupied molecular orbital (LUMO) of MB and the CB edge of the H_*x*_K_1−*x*_NbO_3_ semiconductor^34,56^. Moreover, RhB was selected to investigate the activity of LSPR-promoted carriers and to avoid competitive photon absorption by the dye as evidenced in the absorption spectra showing that a colored glass filter can efficiently exclude eventual self-absorption of RhB (Fig. 3c). The experiments were carried out at room temperature in air. At each 30 min, 2 mL of aliquot was collected from the reactor. The catalysts were separated by centrifugation and the dye solutions were analyzed by UV–Vis spectrophotometry. The concentration evolution of the dye solution was monitored based on the intensity variation in the featured absorption peak of dyes (*λ*_abs_ of RhB and MB are 554 and 664 nm, respectively).

### Photoelectrochemical water splitting characterizations

The measurement was carried out in an undivided either two- or a three-electrode cell configuration, employing either Au@Nb@H_*x*_K_1−*x*_NbO_3_ NPPs or H_*x*_K_1−*x*_NbO_3_ NSs as the working electrode subjected to the irradiation, a Pt coil or Pt foil as the counter electrode and an additional silver/silver chloride reference electrode (Ag/AgCl in 3 M KCl, 0.207 V vs. NHE) was applied for the three-electrode setup. The photoelectrodes were fabricated upon drop-casting 100 μL Au@Nb@H_*x*_K_1−x_NbO_3_ NPP/H_*x*_K_1−*x*_NbO_3_ NS suspension (20 mg NPP/NS in 1 mL hexane) onto an indium tin oxide (ITO) conducting substrate without post-thermal and -chemical treatments. A potentiostat (CH Instruments, CHI 627D) was used for monitoring the photocurrent behaviors as a function of either potential or time, respectively. A simulated AM 1.5G solar light was used as the irradiation source and the light intensity was calibrated to 100 mW/cm (1 sun). The photocurrent-potential plots were obtained with a potential window from –0.25 to +1.3 V (on the Ag/AgCl scale) at a potential sweeping rate of 10 mV/s. The photocurrent-time plots were recorded at 1 V (vs. Ag/AgCl) with different irradiation wavelengths. For the wavelength-dependent measurement, a UV cutoff filter (*λ* > 420 nm) was employed to eliminate UV light, and different colored glasses (*λ* > 610 nm) were used to progressively filter the integral UV to visible light. The incident light was casting on the photoelectrode from the back side through a quartz window and the electrolyte. Argon-purged aqueous solutions containing either 0.5 M Na_2_SO_4_ or 0.5 M Na_2_SO_4_ and 1 M Na(COOH) were used as the electrolytes.

### Data availability

The data that support the findings of this study are available from the corresponding author upon reasonable request.

## Electronic supplementary material

Supplementary Information
