# Supplementary material for: Au@Nb@HxK1-xNbO3 nanopeapods with near-infrared active plasmonic hot-electron injection for water splitting
Source: Nat Commun. 2018 Jan 16;9:232. doi: 10.1038/s41467-017-02676-w (PMC5770448; doi:10.1038/s41467-017-02676-w)
Supplement: Supplementary file 1 — Supplementary Information [file 41467_2017_2676_MOESM1_ESM.pdf]

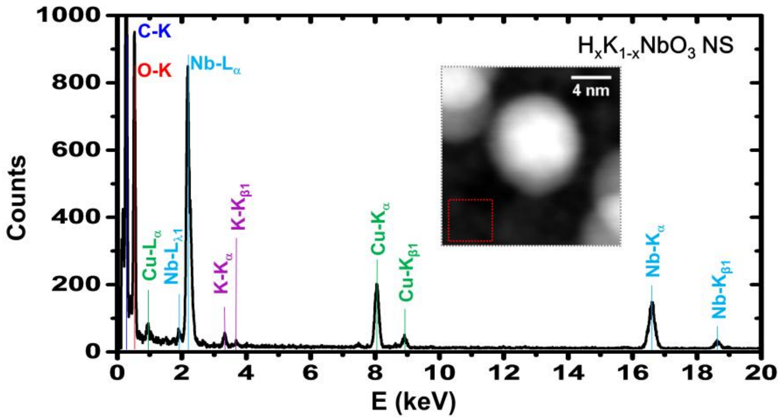

Supplementary Figure 1. EDX spectrum performed in the framed region (rectangle in the inset) to determine the composition of $H_xK_{1\text{-}x}NbO_3$ NSs. Inset: HAADF-STEM image of Au@Nb@$H_xK_{1\text{-}x}NbO_3$ NPPs. The $H_xK_{1\text{-}x}NbO_3$ NSs cannot be recognized due to its low average atomic number compared to the Au@Nb CS-NPs. Abbreviations used: NSs, nanoscrolls; NPPs, nanopeapods.

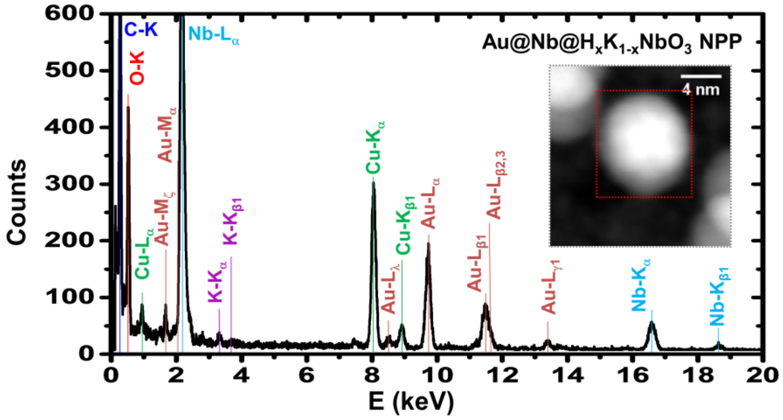

Supplementary Figure 2. EDX spectrum performed at the framed region (rectangle in the inset) to determine the composition of the inner metallic CS-NPs. A chemical composition of $H_{0.03}K_{0.97}NbO_3$ for the semiconductor NS was supposed. Inset: HAADF-STEM image of Au@Nb@$H_xK_{1-x}NbO_3$ NPPs. Abbreviations used: NSs, nanoscrolls; NPPs, nanopeapods; CS-NPs, core-shell nanoparticles.

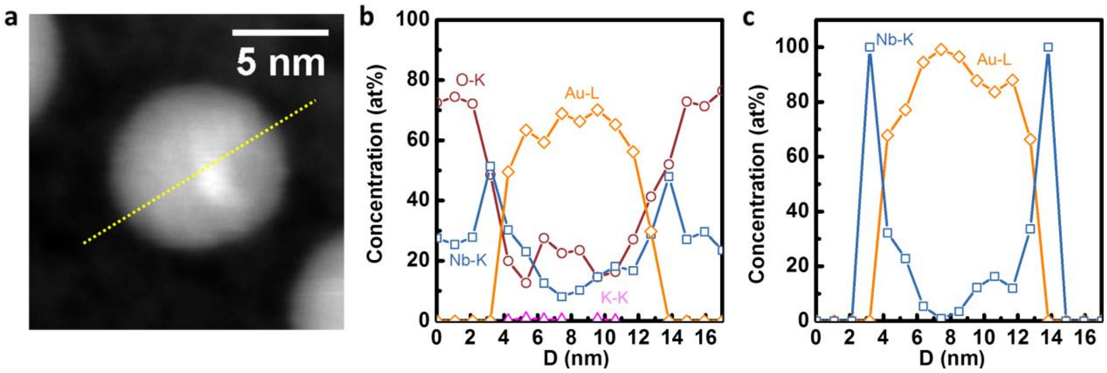

Supplementary Figure 3. Single Au@Nb CS-NP EDXS line scan performed along the yellow dotted line shown in the HAADF-STEM image **(a)**. Compositional profiles for the ensemble **(b)** and single bimetallic CS-NP **(c)**. The latter was derived from subtracting the contribution of the $H_{0.03}K_{0.97}NbO_3$ NS. Abbreviations used: CS-NP, core-shell nanoparticle; NS, nanoscroll.

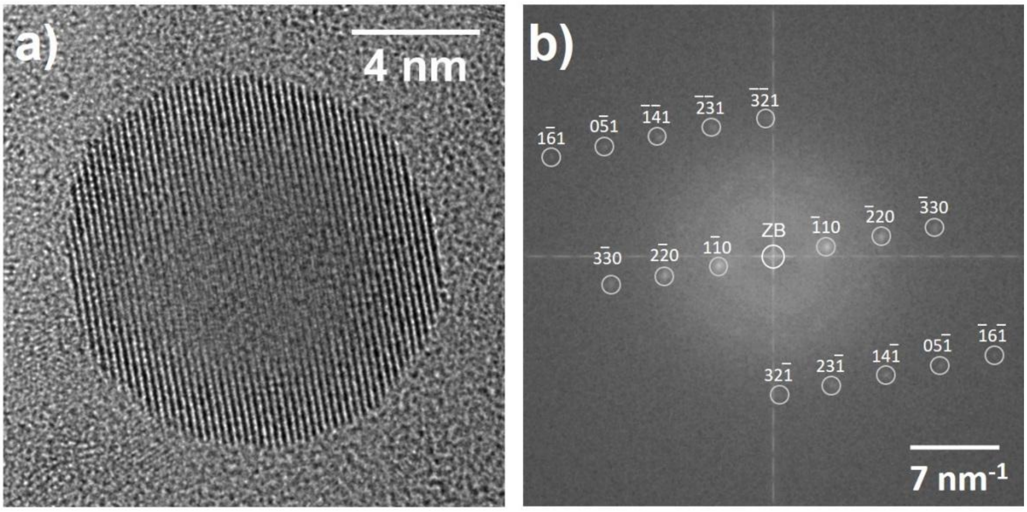

Supplementary Figure 4. Au@Nb CS-NP within a $H_xK_{1-x}NbO_3$ NS. (a) HRTEM image and **(b)** diffractogram of the entire CS-NP in **(a)** and calculated diffraction pattern with Miller indices of bulk *cubic* Nb (space group *Im-3m*, *a* = 3.32 Å) in the [115]-zone axis. The white circle indicates the zero-order beam (ZB). Abbreviations used: CS-NP, core-shell nanoparticle; NS, nanoscroll.

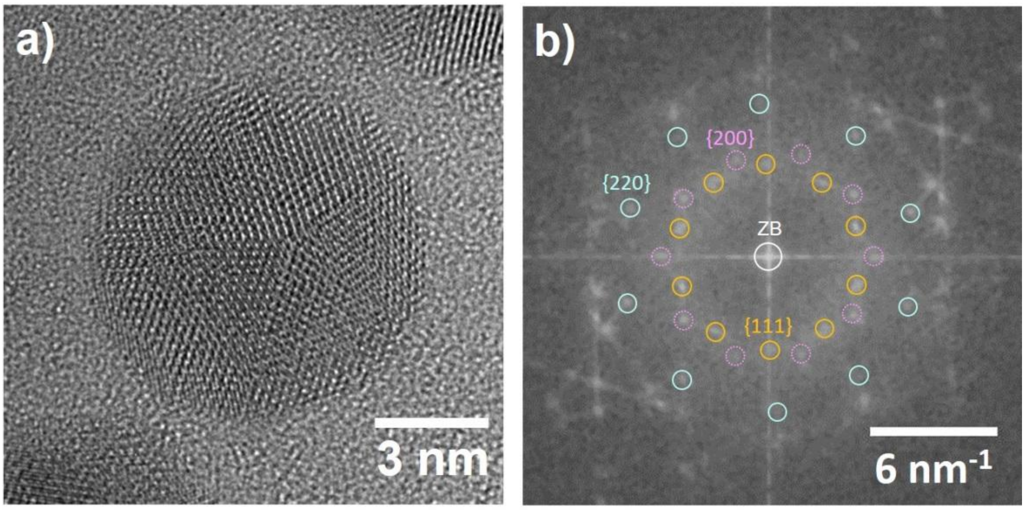

Supplementary Figure 5. Au@Nb CS-NP on a $H_xK_{1-x}NbO_3$ NS. (a) HRTEM image and **(b)** diffractogram of the entire nanoparticle in **(a)** with calculated diffraction pattern with Miller indices for a Au decahedron along its fivefold symmetry axis (Au with space group *Fm-3m*, *a* = 4.08 Å). The white circle indicates the zero-order beam (ZB). Abbreviations used: CS-NP, core-shell nanoparticle; NS, nanoscroll.

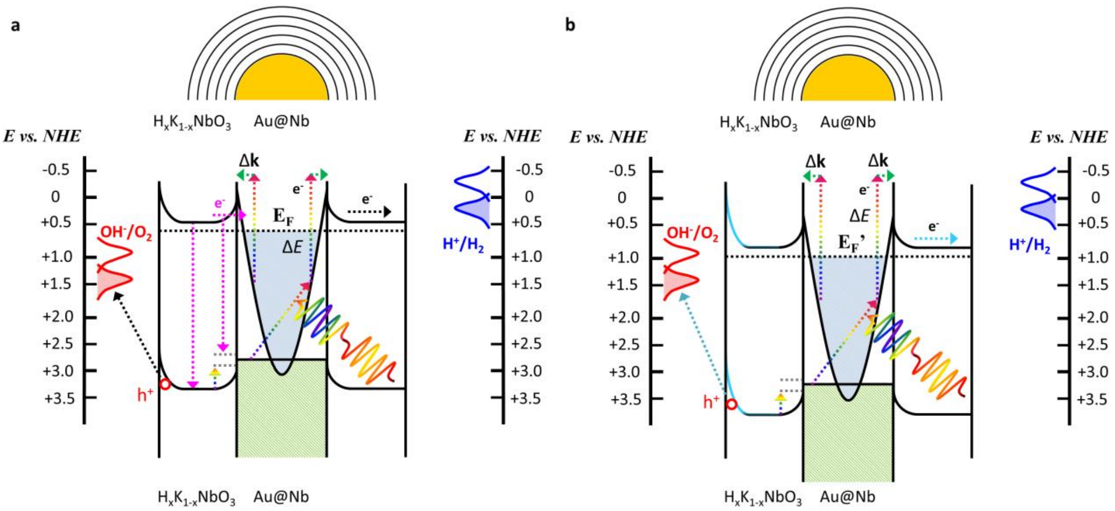

Supplementary Figure 6. External-bias-dependent energetics of the Au@Nb@$H_xK_{1-x}NbO_3$ NPP photoelectrode. (a) Characteristic shunt losses (pink dashed arrows) including electron tunneling, interface and bulk charge recombination impair the carrier collection for photo-electrochemical water splitting in the absence of an anodic bias. **(b)** In the presence of a strong anodic potential, the reinforced band bending within the space-charge layer (light blue curve) effectively rectifies the charge transport (light blue dashed arrows). Colorful oscillations represent the AM1.5G simulated sunlight. Abbreviations used: ΔE, energy change; Δk, momentum change; E_F, Fermi level.

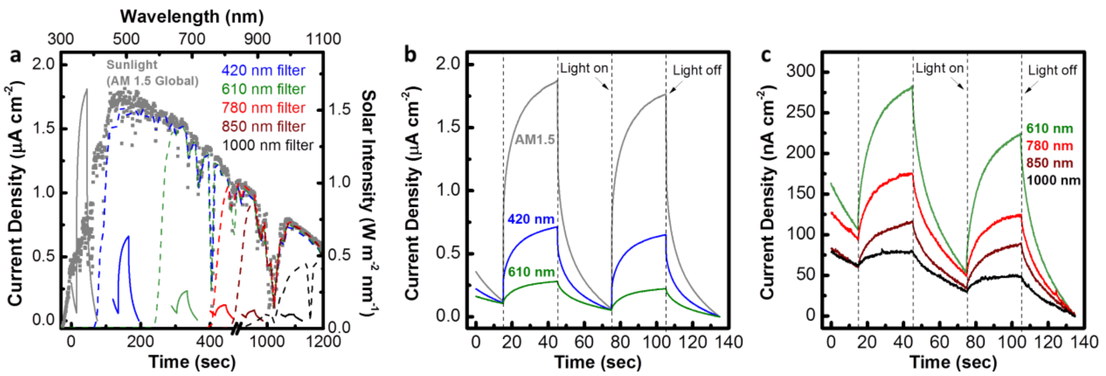

Supplementary Figure 7. Wavelength-dependent photocurrent-time plots of the Au@Nb@$H_xK_{1-x}NbO_3$ NPP photoelectrode in the presence of the Na(COOH) hole scavenger. (a) Diverse long-pass filters were employed to modulate the wavelength of the AM 1.5 G simulated sunlight irradiance. **(b-c)** Corresponding time-dependent photocurrent responses of the Au@Nb@$H_xK_{1-x}NbO_3$ NPP photoelectrode after subtracting the respective baseline originating from the non-faradic charging behavior, and mostly the electrooxidation of the hole acceptor. Abbreviations used: NPPs, nanopeapods.

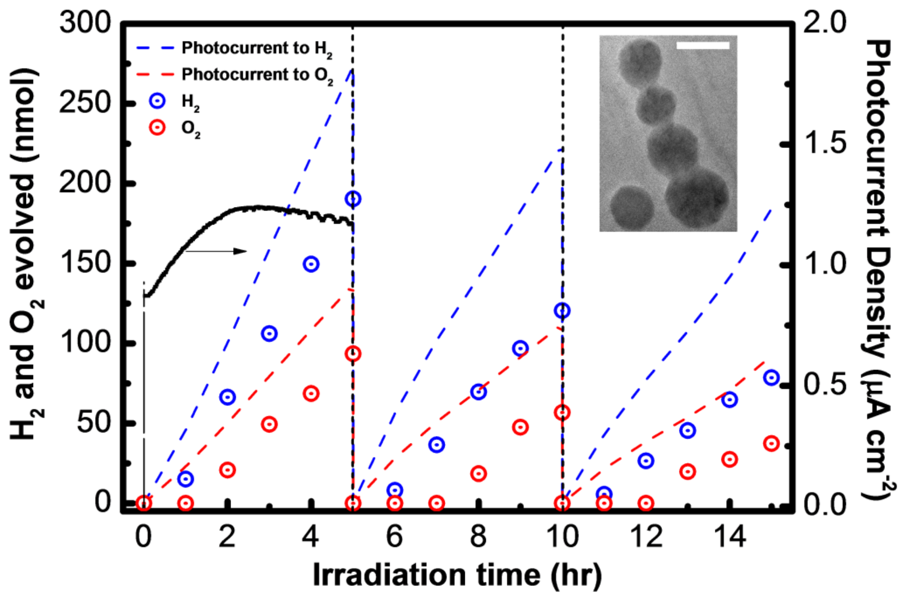

Supplementary Figure 8. Photocurrent transient (baseline subtracted) and the concurrent generation of H_2 and O_2 with the Au@Nb@$H_xK_{1-x}NbO_3$ NPP photoelectrode polarized at +1 V (*vs.* Pt) under AM 1.5 G simulated sunlight illumination. Blue and red dashed lines correspond to the integration of the net photocurrent converted into H_2 and O_2 gases using Faraday's law. Blue and red circled dots respectively correspond to H_2 and O_2 gases measured by gas chromatography during the experiment. SEM image of the postreaction Au@Nb@$H_xK_{1-x}NbO_3$ NPP is shown alongside the data (scale bar: 20 nm). Abbreviations used: NPPs, nanopeapods.

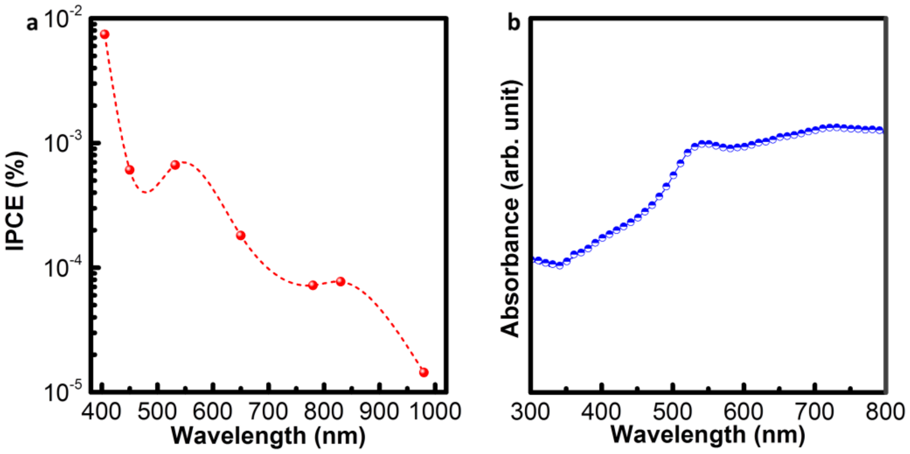

Supplementary Figure 9. Photoelectric conversion efficiency of the Au@Nb@$H_xK_{1-x}NbO_3$ NPP photoelectrode. (a) Photocurrent action spectrum of the Au@Nb@$H_xK_{1-x}NbO_3$ NPP photoelectrode polarized at +1 V (vs. Ag/AgCl) under specific monochromatic irradiations. **(b)** Absorption spectrum of the Au@Nb@$H_xK_{1-x}NbO_3$ NPPs. Abbreviations used: NPP, nanopeapod.

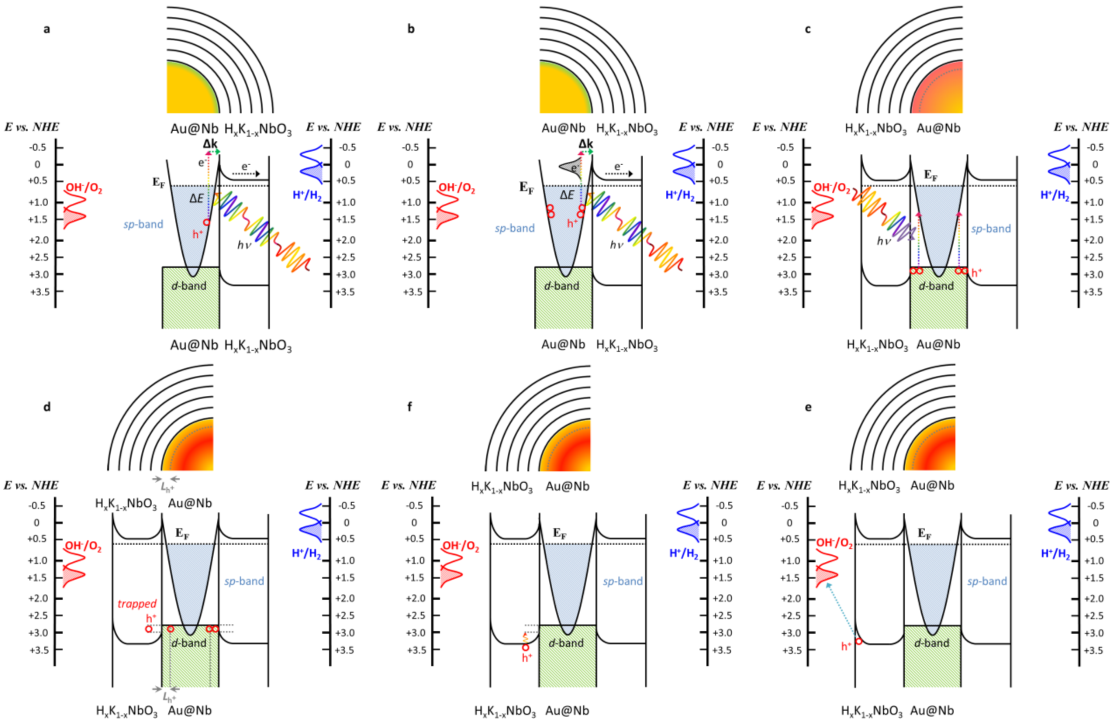

Supplementary Figure 10. Schematic illustration of the Au@Nb@$H_xK_{1-x}NbO_3$ NPP photoelectrode energetics and the cascade charge delivery mechanism under broadband illumination (a-f). In-depth considerations are given in the associated discussion. Abbreviations used: NPPs, nanopeapod; Δk: change in momentum; ΔE: change in energy; L_{h^+}: mean free diffusion length of the hot *d*-band hole.

Supplementary Note 1. Chemical composition and structure of Au@Nb@$H_xK_{1-x}NbO_3$ nanopeapods

The HAADF-STEM image (Supplementary Fig. 1, inset) shows Au@Nb CS-NPs inside $H_xK_{1-x}NbO_3$ nanoscrolls (NSs). The NSs cannot be directly recognized in the HAADF-STEM image due to the large atomic-number difference between the NSs and CS-NPs. EDXS analysis was first performed by analyzing a region without the presence of Au@Nb CS-NPs (Supplementary Fig. 1, framed rectangle of inset) to determine the stoichiometry of the $H_xK_{1-x}NbO_3$ semiconductor.

The quantitative analysis of the EDX spectrum in Supplementary Figure 1 results in a composition of $K_{1\pm0.5}Nb_{25\pm1.0}O_{74\pm1.0}$ corresponding to a stoichiometry of $K_{0.04}Nb_{1.00}O_{2.96}$. Given the diffraction rings in the SAED pattern (see main paper: Fig. 1f) clearly ascribing the cubic $HNbO_3$ crystal phase to the semiconductor sheath and taking into account that H cannot be detected by EDXS, a real composition of $H_{0.96}K_{0.04}NbO_{2.96}$ is concluded. This suggests a significant substitution of K by H during the acid treatment. An average substitution extent x of $x = 0.97$, namely a chemical composition of $H_{0.97}K_{0.03}NbO_3$, was determined based on a statistical evaluation of more than 15 EDX spectra performed at different regions.

On this basis, the formation of a stoichiometric $H_{0.97}K_{0.03}NbO_3$ is supposed in the following. An EDX spectrum of a single NPP (Supplementary Fig. 2) was subsequently recorded by scanning a region with concurrent presence of a metallic CS-NP and a $H_{0.97}K_{0.03}NbO_3$ NS (Supplementary Fig. 2, inset). The latter is not visible in the HAADF-STEM image due to its low average atomic number compared to the CS-NPs. The characteristic X-ray lines belonging to Au (Au–L series: $L_{\alpha1}$, $L_{\alpha2}$, $L_{\beta1}$, $L_{\beta2}$, $L_{\beta3}$, $L_{\beta4}$, $L_{\gamma1}$, $L_{\gamma2}$, L_{υ} and $L_{\lambda1}$ lines) indicates the presence of Au nanoparticles (NPs, Supplementary Fig. 2, inset). The quantification of the EDXS area scan yields a composition of $K_{1.0\pm0.5}Nb_{25.0\pm1.0}O_{62.0\pm1.0}Au_{12.0\pm0.5}$. However, a composition of $K_{1\pm0.5}Nb_{29\pm1}O_{70\pm1}$ was determined in the absence of Au, which corresponds to $K_{0.02}Nb_{1.00}O_{2.46}$. Taking into account that H cannot be detected by EDXS, a real composition of $K_{0.03}H_{0.97}Nb_1O_{2.41}$ is calculated, suggesting an elemental excess of Nb in the ensemble. In other words, a bimetallic CS-NP consisting of Au and Nb in lieu of a solitary Au NP was formed.

The average composition of bimetallic Au@Nb CS-NPs can be calculated by using the average total composition of $K_{1\pm0.5}Nb_{26\pm1}O_{63\pm1.0}Au_{10\pm0.5}$ determined for 10 different CS-NPs by EDXS. The formation of $H_{0.97}K_{0.03}NbO_3$ NSs was considered further, where the Nb atomic concentration is of 1/3 from their corresponding O concentration. After subtracting the NS composition of $K_1Nb_{21}O_{63}$, *i.e.* $H_{0.97}K_{0.03}Nb_{1.00}O_{3.00}$, this

results in an average composition for bimetallic CS-NPs of Nb_5Au_{10}, i.e. $Nb_{0.33}Au_{0.67}$. To determine the spatial distribution of Au and Nb in the bimetallic CS-NPs, an EDXS line scan was performed (Supplementary Fig. 3a, yellow line). The compositional profile of the CS-NPs was plotted as a function of the diameter (*D*). Quantitative analysis was undertaken in a similar way as for the EDXS area scans. In particular, the Nb atomic concentration in the ensemble was distinguished into contributions from the bimetallic CS-NP and $H_{0.97}K_{0.03}NbO_3$ NS. The contribution of the $H_{0.97}K_{0.03}NbO_3$ NS was derived based on a stoichiometric ratio between Nb and O. Namely, the Nb concentration in the $H_{0.97}K_{0.03}NbO_3$ NSs is one-third of the corresponding O atomic concentration. In this way, a heterostructure of Au as the core that is covered by a uniform Nb shell was determined for the CS-NPs upon subtracting the contribution of $H_{0.97}K_{0.03}NbO_3$ NSs from the overall compositional profile (Supplementary Fig. 3b,c). For a clear illustration, the compositional profiles of individual CS-NPs in preference to the ensemble were displayed in the main text.

The structure of the Au@Nb CS-NPs was further investigated by HRTEM. Au NPs within $H_xK_{1-x}NbO_3$ NSs are covered by a continuous shell composed of pure Nb. The entire NP shell in Supplementary Figure 4a is single-crystalline with a bulk *cubic* Nb structure, as demonstrated by the good agreement between its diffractogram (Supplementary Fig. 4b) and the calculated diffraction pattern of the bulk *cubic* Nb in the [115]-zone axis. We note that the experimental diffractogram in Supplementary Figure 4b cannot be attributed to any known Nb_xO_y compound. Detailed EDXS line scans (Supplementary Fig. 1-3) also indicate the presence of a pure Nb shell.

Supplementary Figure 5a shows a HRTEM image of a CS-NP with a Au core, which is covered by a thin incomplete Nb shell. The CS-NP exhibits a multiply twinned structure as demonstrated by the good agreement between its diffractogram (Supplementary Fig. 5b) and the calculated diffraction pattern of a Au decahedron along its fivefold symmetry axis. The thin incomplete Nb shell cannot be recognized in the HRTEM image (Supplementary Fig. 5a) and its diffractogram (Supplementary Fig. 5b). Only the EDXS line-scan analysis shown in Fig. 2d (see main paper) demonstrates the presence of a thin incomplete Nb shell.

Supplementary Note 2. Photoelectrochemical properties of Au@Nb@$H_xK_{1-x}NbO_3$ nanopeapods

External-bias-dependent energetics of the Au@Nb@$H_xK_{1-x}NbO_3$ NPP photoelectrode The (photo)current-potential response was monitored for the Au@Nb@$H_xK_{1-x}NbO_3$ NPP photoelectrode in an aqueous sodium sulfate (Na_2SO_4) electrolyte (pH 6.8) with a three-electrode cell configuration (see main paper: Fig. 4a). Particularly, the potential of the Au@Nb@$H_xK_{1-x}NbO_3$ NPP working photoelectrode was measured against a Ag/AgCl reference electrode.[1] In addition, the current response stemming from the faradaic water (photo)electrolysis flows between the photoelectrode and the Pt counter electrode. Noteworthily, the plasmonic Au@Nb CS-NP nanoantennas and the niobate NSs were concurrently subjected to this external bias. As a consequence, the potential barrier at the Schottky junction between the Au@Nb CS-NPs and the $H_xK_{1-x}NbO_3$ NSs is independent of the applied voltage and exclusively dictated by the energy difference between their respective Fermi levels.
Although two potential barriers are spontaneously established at the Schottky junction between the Au@Nb CS-NPs and the $H_xK_{1-x}NbO_3$ NSs and within the depletion layer between the $H_xK_{1-x}NbO_3$ NSs and H_2O, the dimension of the niobate NS substantially dominates the barrier height at these interfaces in the absence and in the presence of a small external bias (Supplementary Fig. 6a).[2] Thus, undesirable processes – including charge recombination and electron tunneling from the $H_xK_{1-x}NbO_3$ NSs through and over the energy barriers to the Au@Nb CS-NPs and H_2O – markedly short-circuit the photoelectrochemical cell and lead to a lowered photocurrent density (see main paper: Fig. 4a). If a strong bias is applied at the working electrode, the barrier height – particularly within the space-charge layer of the $H_xK_{1-x}NbO_3$ NSs – starts depending on the external voltage (Supplementary Fig. 6b). The reinforced band bending in turn rectifies the carrier transport, wherein most of the photoexcited electrons and holes are swept to the Pt counter electrode and the surface of the Au@Nb@$H_xK_{1-x}NbO_3$ NPP photoanode followed by the injecting into the electrolyte for hydrogen and oxygen generation. This leads to mitigated shunt losses as evidenced by the progressively enhanced photocurrent density with an anodic polarization (see main paper: Fig. 4a).

Modulated reaction kinetics due to the presence of the Na(COOH) hole scavenger The specific advantage of studying the photoelectrochemical property of the Au@Nb@$H_xK_{1-x}NbO_3$ NPPs in the presence of 1 M Na(COOH) is to exclude the perturbation of poor reaction kinetics at the photoelectrode/electrolyte boundary in terms of the formate decomposition known to be thermodynamically and kinetically

more favorable than water oxidation. The elevation and argumentation in the photo-current density in the biased (1 V *vs.* Ag/AgCl) current-time plots (Supplementary Fig. 7) of the Au@Nb@$H_xK_{1-x}NbO_3$ photoelectrodes after the addition of this hole scavenger clearly evidenced the facile photoelectrooxidation of $[COOH]^-$. However, the current transient yet constantly lacked of an anodic and cathodic spike at the light-on/light-off instants, suggesting that the rate-limiting step for the Au@Nb@$H_xK_{1-x}NbO_3$ NPP photoelectrode to transform chemicals is under diffusion control of the photogenerated carrier on the NPPs rather than a kinetic control of the reacting medium.

Water photoelectrolysis by the Au@Nb@$H_xK_{1-x}NbO_3$ NPP photoelectrode for H_2 and O_2 gases evolution Photoelectrolysis of water by the Au@Nb@$H_xK_{1-x}NbO_3$ NPP photoelectrode for H_2 and O_2 generation was substantiated *via* the analysis of the headspace of a custom built, air-tight, single-compartment cell with *in operando* conditions using gas chromatography (GC) (Shimadzu Corporation) (Supplementary Fig. 8). A two-electrode configuration was employed for this cell, in which the Au@Nb@$H_xK_{1-x}NbO_3$ NPPs served as the working photoanode under continuous illumination of AM 1.5 G simulated sunlight over a 5-h period for three cycles. A Pt coil was used as the counter cathode with a potential difference of 1 V to the working photoanode. The amount of evolved H_2 and O_2 gases was determined by taking 100 μL of gas from the headspace of the cell using a syringe. The gas was injected into the gas-sampling loop of the GC in one-hour periodicity. The GC was equipped with a packed MolSieve 5 Å column and a thermal conductivity detector (TCD). Argon (Airgas, ultra high purity) was used as the carrier gas. The quantification of the photoproducts was made while taking the following into account: *i*) side effect of water electrolysis due to the external bias was subtracted from the measurement; *ii*) the dissolution of the evolved gases in the electrolyte was calibrated using Henry's law.[3-5] To the former concern, additional *in operando* analysis of the identical system, but in the absence of any light irradiation was employed to account for the above named side effect. Evidently, the constant evolution of H_2 and O_2 over the 15-h investigation corroborated the performance of the Au@Nb@$H_xK_{1-x}NbO_3$ photoelectrode reasonably stable. The Faradaic efficiencies (η) of the H_2 and O_2 generation on the cathode and the photoanode were calculated by:[6]

$$\eta = \frac{zFn}{Q_{ph}} \quad (1)$$

Herein, F is the Faraday constant (96485 C mol^{-1}), n the mole amount of produced H_2 or O_2, z the number of transferred electrons per mole of evolved gas (i.e., $2e^-$ for H_2 and $4e^-$ for O_2), and Q_{ph} is the integrated photogenerated charge. The derived η resulted in 0.7 for the stoichiometric H_2 and O_2 generation in the first 5-h period, suggesting minor contributions of competitive reactions, which most probably relate to the formation of surface peroxo-species.[6-9]

$$2H_2O \rightarrow H_2O_2 + 2H^+ + 2e^- \tag{2}$$

$$O_2 + 2H^+ + 2e^- \rightarrow H_2O_2 \tag{3}$$

Noteworthily, the SPR band of the resonant nanoantennas was progressively adapted via gradually turning the Au@Nb nanopeas into nanopeanuts (Supplementary Fig. 8, inset) due to the inevitable plasmonic heating effect.[10] Particularly, the nanopeanuts demonstrates a higher aspect ratio than that of spherical nanopeas, leading highly likely to the redshift of longitudinal-mode plasmonic resonance.[10] In consequence, such resonant transition in lieu of deactivation accounts mostly for the minor decays in photocurrent and gas evolving rate over elapsed periods due to the consecutively superimposed charge scatterings in the multi-photon-assisted carrier generation mechanism (Supplementary Note 3) attenuating the photoelectric conversion efficiency (IPCE) at longer wavelengths (Supplementary Fig .9a).[11-12] Likewise, the deteriorated η over the lapse of 15-h water photoelectrolysis stems presumably from the boost of aforementioned charge kinetics (Supplementary Note 3) otherwise favoring those kinetically facile side reactions. Herein, the IPCE was derived via dividing the number of photogenerated electrons by the number of incident monochromatic visible (VIS) and near-infrared (NIR) photons with the wavelength spanned from about 400 nm up to nearly 1000 nm, a width of ±5 nm and an intensity ranging from 20 to 50 mW cm^{-2}, respectively. Prima facie, the photocurrent action spectrum of the Au@Nb@$H_xK_{1-x}NbO_3$ NPP photoelectrode (Supplementary Fig. 9a) tracks the transverse-mode LSPR band and the longitudinal-mode LSPR plateau that are manifested in the absorption spectrum (Supplementary Fig. 9b) of the Au@Nb@$H_xK_{1-x}NbO_3$ NPP reasonably well. Moreover, the IPCE progressively lowers from the blue to the red regime of the action spectrum with values substantially of the order of a few to few tens of millionth, which in turn gives rise to the photocurrent density at a level of few tenths to a few nanoamperes (nA cm^{-2}) at individual monochromatic wavelengths under full solar illumination with a fluency of 100 mW cm^{-2}. Given the VIS and NIR light exhibiting a wavelength span of hundreds to thousands of nanometers in diffusive sunlight, the integrated photocurrent density

over this interval is up to the order of few tenths of microamperes (μA cm^{-2}). In fact, this is in excellent agreement with the order of magnitude of the photocurrent transients of the Au@Nb@$H_xK_{1-x}NbO_3$ NPP photoelectrode as derived under sunlight illumination exclusive of UV light (see main paper: Fig. 4c).

Supplementary Note 3. Non-Linear plasmonics in Au@Nb@$H_xK_{1-x}NbO_3$ nanopeapods

Kinetic model of the nonlinear plasmonics of the Au@Nb@$H_xK_{1-x}NbO_3$ nanopeapods A kinetic scheme describing the elementary steps of the cascade mechanism for the Au@Nb@$H_xK_{1-x}NbO_3$ NPP photoelectrode under AM 1.5 G simulated sunlight illumination (see main paper: Fig. 4e) includes: *i*) the hot electron photogeneration on Au@Nb CS-NPs *via* an intraband transition (*sp→sp*), *ii*) the electron/hole (e^-/h^+) pair photogeneration on $H_xK_{1-x}NbO_3$ NSs *via* an interband transition, and *iii*) the electron injection across the Schottky interface followed by restoring the *sp*-band vacancy on the Au@Nb CS-NPs, while *iv*) the hole in the valence band (VB) of the $H_xK_{1-x}NbO_3$ NSs sweeps to the solid-liquid phase boundary, where it is injected into water (H_2O) for oxygen (O_2) generation. The specific reactions are shown below:

$$h\nu + Au@Nb \xrightarrow{k_1F_{h\nu}} e_{sp}^- + h_{sp}^+ \qquad (4)$$

$$h\nu + H_xK_{1-x}NbO_3 \xrightarrow{k_2F_{h\nu}} e_{CB}^- + h_{VB}^+ \qquad (5)$$

$$e_{CB}^- + h_{sp}^+ \xrightarrow{k_3} heat \qquad (6)$$

$$h_{VB}^+ + H_2O \xrightarrow{k_4} \tfrac{1}{2}O_2 + 2H^+ \qquad (7)$$

Herein, $F_{h\nu}$ is the photon flux impinging on the Au@Nb@$H_xK_{1-x}NbO_3$ NPP photoelectrode, h_{sp}^+ and e_{sp}^- are the hole and the electron at the *sp*-band of the Au@Nb CS-NP, h_{VB}^+ and e_{CB}^- are the hole and electron in the valence and conduction band (CB) of the $H_xK_{1-x}NbO_3$ NSs.[7,13,14] Such kinetic model suggested a linear dependence of the hole flux on the light intensity for the Au@Nb@$H_xK_{1-x}NbO_3$ photoelectrode in the absence of additional charged carrier losses in the bulk of Au@Nb@$H_xK_{1-x}NbO_3$ according to:

$$-\frac{d[h_{VB}^+]}{dt} = -\frac{d[H_2O]}{dt} = k_2k_4[H_2O]F_{h\nu} \qquad (8)$$

This dependency turns into a square-root-type relation for the charge recombination in the bulk of the Au@Nb@$H_xK_{1-x}NbO_3$ NPP.[13,15]

$$-\frac{d[h_{VB}^+]}{dt} = -\frac{d[H_2O]}{dt} = k_4[H_2O](\frac{k_2k_3 - k_1k_6}{k_3k_5})^{\frac{1}{2}}F_{h\nu}^{\frac{1}{2}} \qquad (9)$$

$$h_{VB}^{+} + e_{CB}^{-} \xrightarrow{k_5} heat \quad (10)$$

$$h_{VB}^{+} + e_{sp}^{-} \xrightarrow{k_6} heat \quad (11)$$

Supplementary Eqn. (9) was derived based on the assumptions of: *i*) a fast equilibrium between the charge-carrier formation (*i.e.*, Supplementary steps 4 and 5) and the charge-carrier recombination (*i.e.*, Supplementary steps 6, 10, and 11), and *ii*) the steady-state approximation of $[e_{sp}^{-}] \approx [h_{sp}^{+}]$ = constant as well as $[e_{CB}^{-}] \approx [h_{VB}^{+}]$ = constant. Herein, the rate of the H_2O photooxidation *(i.e.*, Supplementary step 7) expressed by the anodic photocurrent density (see main paper: Fig. 4d) is employed to faithfully reflect the hole flux reaching the surface of the Au@Nb@$H_xK_{1-x}NbO_3$ NPP photoelectrode. Noteworthy, such description holds only *i*) in the absence of surface recombinations, and *ii*) at low $F_{h\nu}$ characterized by $[h_{VB}^{+}] << [H_2O]$.[7] To meet these criteria, the overall photocurrent-time response of the Au@Nb@$H_xK_{1-x}NbO_3$ NPP photoelectrode – coupled with an aqueous sodium sulfate (Na_2SO_4) electrolyte – was collected with an anodic bias of 1 V (*vs.* Ag/AgCl). A very important result of the strong anodic bias relates to the significantly reinforced potential barrier (Φ_S) within the space-charge layer, leading to a virtually "frozen out" charge recombination at the electrode-electrolyte interface owing to the majority electrons that are depleted in the space-charge region as suggested by the Boltzmann expression.[7,16-19]

$$n_s = n_b \exp\left(-\frac{e\phi_s}{kT}\right) \quad (12)$$

In this equation, n_s and n_b represent the surface and bulk density of electrons, e is the electronic charge, k the Boltzmann constant, and T is the temperature. In other words, the detected photocurrent density substantially describes the hole flux that participates in the Faradaic water photooxidation process. Moreover, the photocurrent transients were collected under exposure of the broadband irradiation with the intensity not more than "one sun" to ensure the reaction rate to be mostly dictated by the hole flux in preference to the water oxidation kinetics.[7]

To our surprise, the initial photocurrent shoot (I_{in}) obtained at the light-on moment manifests a superlinear dependency on the intensity of AM 1.5 G simulated sunlight (see main paper: Fig. 4f). This suggests an influence of non-linear optical effect on the process of the hole photogeneration *via* the Au@Nb@$H_xK_{1-x}NbO_3$ NPPs. In particular, such response arises exclusively from plasmonic excitation, as evidenced by the quasi-linear behavior of the $H_xK_{1-x}NbO_3$ NS photoelectrode without any plasmonic Au@Nb nanoantennas (see main paper: Fig. 4h). Most remarkably, the

pseudo-quadratic correlation indicates a two-photon absorption of the centrosymmetric Au@Nb CS-NPs *via* a third-order photon-photon interaction, which is in accordance with the characteristic non-linearity of the two sequential one-photon absorption process.[14,20] The elementary steps of the overall kinetic model describing the cascade mechanism are displayed below (see main paper: Fig. 4g).

$$h\nu + Au@Nb \xrightarrow{k_1 F_{h\nu}} e^-_{sp} + h^+_{sp} \tag{4}$$

$$h\nu + h^+_{sp} \xrightarrow{k_7 F_{h\nu}} h^+_d \tag{13}$$

$$h^+_d + SS \xrightarrow{k_8} SS^+ \tag{14}$$

$$SS^+ + \hbar\omega \xrightarrow{k_9 F_{\hbar\omega}} h^+_{VB} \tag{15}$$

$$h^+_{VB} + H_2O \xrightarrow{k_5} \tfrac{1}{2}O_2 + 2H^+ \tag{7}$$

Herein, h_d^+ is the hole in the *d*-band of the Au@Nb CS-NP, $F_{\hbar\omega}$ is the phonon flux spontaneously emitted from the lattice vibration, and SS^+ is the hole at the mid-gap surface state of the $H_xK_{1-x}NbO_3$ NSs at the Schottky interface.[14] Noteworthy, the *sp*-band vacancy stemming from the intraband transition (*i.e.*, Supplementary step 4) is substantially restored *via* the interband transition (*d*→*sp*) of the Au@Nb CS-NPs in this formulism to in turn photogenerate the energetic *d*-band hole (*i.e.*, Supplementary step 13). The prominent role of such non-linear optical process mostly results from the localized surface plasmons (LSPs) that boost the local electromagnetic field that allows the inherently weak non-linear effect to be significantly enhanced.[14,20] Moreover, the inferior charge-transport kinetics of the $H_xK_{1-x}NbO_3$ NSs, wherein drift and diffusion of the electrons (e_{CB}^-) are perpendicular to the basal lattice plane with higher interlamellar distances than in the bulk counterpart, reduces the probability of a e_{CB}^- and h_{sp}^+ recombination.[2,21,22] Afterwards, the hot *d*-band hole is extracted to the VB of the $H_xK_{1-x}NbO_3$ NSs (*i.e.*, Supplementary step 15), wherein the mid-gap surface state (*SS*) at the Schottky junction serves as an important relay (*i.e.*, Supplementary step 14). With *SS* mainly stemming from the stepwise nano-texturization, the structural defects lead to numerous mid-gap electronic levels that are generally located at shallow energy positions close to the VB edge.[23] Consequently, the thermal transfer of the hole from those trapped sites to the VB readily takes place, as evidenced by the photocurrent transient of the $H_xK_{1-x}NbO_3$ NS photoelectrode under the irradiation with sub-bandgap-energy integral VIS-NIR light (see main paper: inset in Fig. 4h). The photocurrent response is presumably attributed to a serial process beginning with an electron excitation from a discrete surface-defect level to the continuous band state and

vice versa. Afterwards, the h^+/e^- at the mid-gap energy state ends in entering the VB/CB *via* thermal transition, as evidenced by the linear correlation likewise established between the measured photocurrent and the light intensity (see main paper: Fig. 4h). Eventually, h_{VB}^+ was delivered to the solid-to-liquid boundary and thereat interacts with H_2O (*i.e.*, Supplementary step 7).

The total kinetic analysis comprises a quadratic dependence

$$-\frac{d[h_{VB}^+]}{dt} = -\frac{d[H_2O]}{dt} = k_1 k_4 k_7 k_8 k_9 [SS][H_2O] F_{\hbar\omega} F_{h\nu}^2 \qquad (16)$$

of the hole flux on the light intensity for the $Au@Nb@H_xK_{1-x}NbO_3$ NPP photoelectrode in the absence of charge-carrier losses and scattering in the bulk of the $Au@Nb@H_xK_{1-x}NbO_3$ NPP. In contrast, the measured sub-quadratic behavior indicates that the charge-carrier decay should not be overlooked (see main paper: Fig. 4f). In particular, the charge recombination in the bulk of the $H_xK_{1-x}NbO_3$ NSs is virtually ruled out, provided that the featured quasi-linear correlation of the $H_xK_{1-x}NbO_3$ NS photoelectrode implies that such losses are efficiently suppressed (*see main paper: Fig. 4h*). This can be attributed to the depletion region developed by the applied bias that nearly overwhelms the overall niobate NS in terms of the characteristic width (W) and that is ruled by the extent of the band bending (Φ_s).

$$W = \sqrt{\frac{2\varepsilon\varepsilon_0\phi_s}{eN_D}} \qquad (17)$$

Herein, ε_0 is the permittivity of free space, N_D and ε are the charge carrier density and the dielectric constant of the $H_xK_{1-x}NbO_3$ NSs.[16,17] In other words, the charge carriers are mostly lost in the process of surface plasmon resonance (SPR) excitation, wherein the charge scattering and recombination are the major contributors.[14,24,25] Particularly, the scattering of h_{sp}^+ and h_d^+ proceeds at the rates of $[h_{sp}^+]/\tau_{sp}$ and $[h_d^+]/\tau_d$, wherein τ_{sp} and τ_d represent the relaxation time of the *sp*- and *d*-band holes. Given that τ is on a femtosecond timeframe, h_{sp}^+ and h_d^+ very likely undergo scattering events prior to the recombination.

$$e_{sp}^- + h_{sp}^+ \xrightarrow{k_{10}} heat \qquad (18)$$

$$e_{sp}^- + h_d^+ \xrightarrow{k_{11}} heat \qquad (19)$$

The inclusion of the aforementioned carrier losses in the kinetic interpretation give a correlation of the photooxidation rate of H_2O to the sub-second-order incident light intensity:

$$-\frac{d[H_2O]}{dt} = k_4 k_8 k_9 [SS][H_2O] F_{h\omega} \frac{\frac{-k_7}{2k_{10}\tau_{sp}} F_{h\nu} + \frac{k_7}{2k_{10}} \sqrt{\frac{1}{\tau_{sp}^2} + 4k_1 k_{10} F_{h\nu}} F_{h\nu}}{\{k_8[SS] + \frac{1}{\tau_d} - \frac{k_{11}}{2k_{10}\tau_{sp}}\} + \frac{k_{11}}{2k_{10}} \sqrt{\frac{1}{\tau_{sp}^2} + 4k_1 k_{10} F_{h\nu}}} \quad (20)$$

In particular, Supplementary Eqn. 20 is derived based on the approach of the probability of the recombination between e_{sp}^- and h_{sp}^+ (*i.e.*, Supplementary step 18) predominating over that between e_{sp}^- and h_d^+ (*i.e.*, Supplementary step 19) in terms of $[h_{sp}^+] >> [h_d^+]$. This was inferred from the higher electron density (n_{sp}) in the *sp*-band, which is essential for the significantly intraband transition (*i.e.*, Supplementary step 4), in comparison to the density of created *sp*-band vacancies ($n_{h_{sp}^+}$) that are necessary for the interband transition (*i.e.*, Supplementary step 13). On such basis, $[e_{sp}^-] = [h_{sp}^+] + [h_d^+] \approx [h_{sp}^+]$ was concluded. In addition, the assumptions of *i*) a fast equilibrium between charge-carrier formation (*i.e.*, Supplementary steps 4 and 13), scattering and recombination (*i.e.*, Supplementary steps 18 and 19), and *ii*) the steady-state approximation of $[e_{sp}^-] \approx [h_{sp}^+]$ = constant were likewise applied. This conclusion is in good agreement with the experimental observations (see main paper: Fig. 4f). Most importantly, the merit of such coincidence particularly manifests in endowing further investigations regarding the dependence of the charged carrier losses on the wavelength of the incident light with physical meaning, which, however, is beyond the scope of this study, and which therefore will be presented elsewhere.[14,24,25]

Energetics of the nonlinear plasmonics of the Au@Nb@$H_xK_{1-x}NbO_3$ nanopeapods Regarding the cascade-like carrier delivery mechanism to describe the transient photocurrent behavior of the Au@Nb@$H_xK_{1-x}NbO_3$ NPPs under broadband illumination, the course of the process always begins with the first hot electron generated on the Au@Nb plasmonic nanoantenna by an intraband transition (*sp→sp*) due to a surface plasmon resonance (SPR) excitation (see main paper: Fig. 4g). Such indirect transition require a change not only in energy (ΔE) but also in momentum (Δk), wherein ΔE is exclusively harvested from the decay of the surface plasmon polaritons (SPPs) that are generated by the resonant photon, whereas Δk is more likely overcome with support of a phonon or an imperfection (Supplementary Fig. 10a).[25] Such defect-assisted hot carrier generation mechanism (including hot electron and hot hole) suggests energetic charges that are preferentially formed at the margin of the

sub-10-nm-sized Au@Nb CS-NP with the numerous, intrinsic imperfections available at the boundary to the $H_xK_{1-x}NbO_3$ NSs in the NPPs. Such spatially selective distribution as well as the 3D Schottky junction stem from the specific NPP-type structure that favors hot electron injection from the metal to the semiconductor,[26] as distinctly evidenced in the Au and Nb L_3-edge X-ray absorption spectra (see main paper: Fig. 4j,k).

The efficiency of the charge transfer additionally benefits from the excellent electron-uptake properties of the $H_xK_{1-x}NbO_3$ NSs since an abundant density of states (DOS) is accessible in the conduction band that is primarily established of Nb^{5+} with d^0 configuration ([Kr]$4d^05s^0$).[27,28] In general, the process is known to occur on a femtoseconds timeframe for plasmonic d^0 metal oxide composites.[28] Moreover, electron delivery is devoid of energy dissipation in terms of the characteristic mean free path (MFP) up to an order of few tens of nanometers, which originates from the free-electron-like nature of the *sp*-band.[24] Consequtive to this process is another SPR excitation via the next incoming photon that produces additional hot carriers, including either *i*) an energetic *sp*-band electron-hole pair *via* indirect intraband transition, or *ii*) a lukewarm *sp*-band-electron coupled with a hot *d*-band hole *via* direct interband transition ($d \rightarrow sp$). In the first few femtoseconds, the former process predominates in terms of the appreciable DOS above the Fermi level[25] and leads to rapid accumulation of hot *sp*-band holes with energies below the Fermi level (Supplementary Fig. 10b).[11] In particular, the external bias herein played an important role in effectively preventing the hot electrons that were already extracted to $H_xK_{1-x}NbO_3$ from drifting back to the Au@Nb CS-NPs for hole-annihilation by immediately shuttling to the Pt counter electrode.[16,17] The quantitative surge in the *sp*-band-electron vacancies in turn reinforces the probability of the interband transition, wherein the tepid *sp*-band holes are restored to generate the hot *d*-band holes (Supplementary Fig. 10c).

Noteworthy, the energetic *sp*-band electrons and the *d*-band holes are formed in the above sequential process (designated two sequential one-photon absorption),[14,29] which requires only a few photons with energies well below the threshold in a single-photon-triggered interband transition.[24,25] Such mechanism should not be overlooked in the Au@Nb@$H_xK_{1-x}NbO_3$ NPPs in view of the peculiar SPR absorption spanned from visible to NIR light and allowing an army of electron vacancies that are created under broadband sunlight in the free-electron-like *sp*-band (see main paper: Fig. 2h). Moreover, *i*) suppressed electron-electron scattering, wherein the hot *sp*-band electron with energy above the Fermi level (ΔE-E_F) interacted with another with energy below the Fermi level to generate three charges (two electrons and one hole) with equipartitioned energies of (ΔE-E_F)/3, due to the instant electron extraction by

$H_xK_{1-x}NbO_3$ from Au@Nb, and *ii*) minor electron-phonon scattering owing to finite DOS available in the *sp*-band below the Fermi level otherwise ensured the energy conservation of the tepid *sp*-band holes to permit the subsequent interband transition.[25,28,29] Nevertheless, unlike the indirect intraband transition, the absence of requisite momentum change (Δk) in this direct transition suggested the delocalization of the *d*-band holes.[11,25] Given the bound nature of *d*-band owing to plenty DOS wherein, the featured MFP of *d*-band carriers was of order very few nanometres.[24,25,30] Taken together, only those *d*-band holes formed within the first few nanometers near the surface of the Au@Nb CS-NP highly likely traverse the boundary into the $H_xK_{1-x}NbO_3$ NSs without energy dissipation (Supplementary Fig. 10d). Such effect is nevertheless highly important due to the enormous surface-area-to-volume ratio of the sub-10-nm-sized Au@Nb CS-NPs (9 nm in average), accounting for more than three tenths of the overall hot *d*-band holes (≥1 nm of MFP for the holes on the *d*-band edge) that presumably enter the niobate.[24] In this hole uptake, the mid-gap surface states of the $H_xK_{1-x}NbO_3$ NSs at the interface with the Au@Nb CS-NPs – which mainly originating from the stepwise nano-texturization – function as important relays,[7,31] provided that such process proceeds isoenergetically.[17] Noteworthy, these highly defect-associated mid-gap electronic levels are generally identified with a shallow energy position close to the VB edge.[23] Consequently, a thermal transfer of the hole from those trapped sites to the VB readily takes place, as manifested by the obvious photocurrent transients of the $H_xK_{1-x}NbO_3$ photoelectrode under irradiation with sub-bandgap integral VIS-NIR light (see main paper: inset in Fig. 4h). The photocurrent response is presumably attributed to a serial process beginning with an electron excitation from discrete surface-defect levels to a continuous band state and *vice versa*. Afterwards, the holes/electrons at the mid-gap energy state end up in entering the VB/CB via thermal transition (Supplementary Fig. 10e). With a strong upward band bending in the $H_xK_{1-x}NbO_3$ NSs, that is oriented towards the water phase due to the external bias, these charges are successfully swept to the $H_xK_{1-x}NbO_3/H_2O$ interface and injected into the electrolyte for oxygen generation (Supplementary Fig. 10f).[16,17] Taken together, the hysteresis of the sequential interband transition for the hot *d*-band hole generation and the subsequent cascade delivery are responsible for the distortion in the transient photocurrent response of the Au@Nb@$H_xK_{1-x}NbO_3$ NPP photoanode under a broadband illumination (see main paper: Fig. 4c).

Supplementary Note 4. Near-infrared active plasmonic hot-electron injection for water splitting

For the photoelectrolytic process of water splitting for hydrogen-fuel generation via a semiconductor, the overall Gibbs free energy (ΔG) of ~2 eV comprises: *i*) the standard ΔG^0 of 1.23 eV for the overall reaction per electron ($2H_2O \rightarrow 2H_2 + O_2$), *ii*) an entropic energy loss ($T\Delta S$) associated to the generation of photoexcited carriers, and *iii*) a kinetic energy loss due to the overpotentials (η) of oxygen and hydrogen generation.[32] Such threshold energy sets up the upper limit of the photon wavelength (λ ~ 610 nm) employed for single-photon-driven water splitting. The application of plasmonic antennas in such one-photon process – aiming at endowing typical UV-active semiconductors with additional SPR-initiated hot electrons to promote the photon-to-fuel conversion efficiency – can hardly work very well in view of the serious energy dissipation *via* the strong electron-electron scattering in the visible range.[25] Nonetheless, such frustration otherwise highlights the merits of subduing NIR photons by plasmonic antennas for fuel production in terms of the highly frequency-dependent electron-electron scattering playing a progressively minor role at longer photon wavelength.[24] As a consequence, most of the SPR-mediated hot carriers generated in the scenario of NIR-light-triggered water splitting *via* a multi-photon-assisted mechanism – according to the energy conservation law – preserve substantial energy until ending in the hydrogen fuel.[11,12] Most importantly, this multi-photon process allows more photons to be available to a plasmonic device from broadband sunlight, rendering further advances in conversion efficiency readily conceivable in the near future.[17]

Supplementary Methods.

Quantification and line scan analyses via energy dispersive X-ray spectroscopy (EDXS) High-angle annular dark-field scanning transmission electron microscopy (HAADF-STEM) combined with EDXS was used to investigate the chemical composition of the Au@Nb core-shell nanoparticles (CS-NPs) and the tubular $H_xK_{1-x}NbO_3$ envelops in the Au@Nb@$H_xK_{1-x}NbO_3$ nanopeapods (NPPs). The experiments were performed with a FEI Osiris ChemiSTEM microscope at 200 kV acceleration voltage, which is equipped with a Bruker Quantax system (XFlash detector) for EDXS. EDX spectra were quantified with the FEI software package "TEM imaging and analysis" (TIA) version 4.7 SP3. Using TIA, element concentrations were calculated on the basis of a refined Kramers' law model, which includes corrections for detector absorption and background subtraction. For this purpose, standard-less quantification, *i.e.*, by means of theoretical sensitivity factors, without thickness correction was applied. EDX spectra obtained during scanning of a rectangular area within single NPPs were used to determine their average chemical composition. The concentration profiles of different chemical elements within a NPP were determined from EDX spectra measured along a line-scan that passes through its center. EDXS line-profiles were recorded by applying a drift-correction routine via cross correlation of several images, which yields a local precision better than 1 nm. The drift-corrected EDXS line-profiles were taken with a probe diameter of 0.5 nm and a distance of about 1 nm between two measuring points. The quantification of EDX spectra was performed by using the Nb–*K* (Nb–*K* series: $K_{\alpha 1}$, $K_{\alpha 2}$ and $K_{\beta 1}$ lines), O–*K* (*K* line), K–*K* (K–*K* series: $K_{\alpha 1}$ and $K_{\beta 1}$ lines) and Au–*L* (Au–*L* series: $L_{\alpha 1}$, $L_{\alpha 2}$, $L_{\beta 1}$, $L_{\beta 2}$, $L_{\beta 3}$, $L_{\beta 4}$, $L_{\gamma 1}$, $L_{\gamma 2}$, L_{υ} and $L_{\lambda 1}$ lines) series. We emphasize that H cannot be detected by EDXS and that X-ray lines of copper (Cu–*K* series: $K_{\alpha 1}$, $K_{\alpha 2}$ and $K_{\beta 1}$ lines and Cu–*L* series: $L_{\alpha 1}$, $L_{\alpha 2}$ and $L_{\beta 1}$ lines) from the grid and carbon (C–*K* line) from the amorphous carbon substrate were also observed in all EDXS spectra. Moreover, EDXS elemental maps of Nb (Nb–$K_{\alpha 1}$ line), O (O–*K*), K (K–$K_{\alpha 1}$) and Au (Au–$L_{\alpha 1}$) were recorded and used to investigate their distribution. The maps were analyzed by using the ESPRIT software (version 1.9) from Bruker.

Supplementary References

[1] Bard, A. J. & Faulkner, L. R. *Electrochemical Methods Fundamentals and Applications.* Wiley, Hoboken, NJ, USA, 2001.

[2] Osterloh, F. E. Inorganic nanostructures for photoelectrochemical and photocatalytic water splitting. *Chem. Soc. Rev.* **42**, 2294-2320 (2013).

[3] Kim, T. W. & Choi, K. -S. Nanoporous $BiVO_4$ Photoanodes with Dual-Layer Oxygen Evolution Catalysts for Solar Water Splitting. *Science* **343**, 990-994 (2014).

[4] Park, H. S., Lee, H. C., Leonard, K. C., Liu, G., & Bard, A. J. Unbiased Photoelectrochemical Water Splitting in Z-Scheme Device Using W/Mo-Doped $BiVO_4$ and $Zn_xCd_{1-x}Se$. *ChemPhysChem* **14**, 2277-2287 (2013).

[5] Ueno, K., Oshikiri, T. & Misawa, H. Plasmon-Induced Water Splitting Using Metallic-Nanoparticle-Loaded Photocatalysts and Photoelectrodes. *ChemPhysChem* **17**, 199-215 (2016).

[6] Kim, H. -I., Monllor-Satoca, D., Kim, W. & Choi, W. N-doped TiO_2 nanotubes coated with a thin TaO_xN_y layer for photoelectrochemical water splitting: dual bulk and surface modification of photoanodes. *Energy Environ. Sci.* **8**, 247-257 (2015).

[7] Salvador, P. Kinetic Approach to the Photocurrent Transients in Water Photoelectrolysis at n-TiO_2 Electrodes. 1. Analysis of the Ratio of the Instantaneous to Steady-State Photocurrent. *J. Phys. Chem.* **89**, 3863-3869 (1985).

[8] Hisatomi, T., Kubota J. & Domen, K. Recent advances in semiconductors for photocatalytic and photoelectrochemical water splitting. *Chem. Soc. Rev.* **43**, 7520–7535 (2014).

[9] Jones, J. E., Hansen, L. D., Jones, S. E., Shelton, D. S. & Thorne, J. M. Faradaic efficiencies less than 100% during electrolysis of water can account for reports of excess heat in “cold fusion” cells. *J. Phys. Chem.* **99**, 6973–6979 (1995).

[10] Zhang, X., Chen, Y. L., Liu, R. S. & Tsai, D. P. Plasmonic photocatalysis. *Rep. Prog. Phys.* **76,** 046401 (2013).

[11] Nishijima, Y. et al. Near-infrared plasmon-assisted water oxidation. *J. Phys. Chem. Lett.* **3**, 1248-1252 (2012).

[12] Nishijima, Y., Ueno, K., Yokota, Y., Murakoshi, K. & Misawa, H. Plasmon-Assisted Photocurrent Generation from Visible to Near-Infrared Wavelength Using a Au-Nanorods/TiO_2 Electrode. *J. Phys. Chem. Lett.* **1**, 2031-2036 (2010).

[13] Thompson, T. L. & Yates, J. T. Jr. Monitoring Hole Trapping in Photoexcited TiO_2(110) Using a Surface Photoreaction. *J. Phys. Chem. B* **109**, 18230-18236 (2005).

[14] Biagioni, P. *et al.* Dependence of the two-photon photoluminescence yield of gold nanostructures on the laser pulse duration. *Phys. Rev. B* **80**, 045411 (2009).

[15] Ingram, D. B. & Linic, S. Water Splitting on Composite Plasmonic-Metal/Semiconductor Photoelectrodes: Evidence for Selective Plasmon-Induced Formation of Charge Carriers near the Semiconductor Surface. *J. Am. Chem. Soc.* **133**, 5202-5205 (2011).

[16] Bard, A. J. Photoelectrochemistry and Heterogeneous Photocatalysis at Semiconductors. *J. Photochem.* **10**, 59-75 (1979).

[17] Nozik, A. J. Photoelectrochemistry: Applications to Solar Energy Conversion. *Ann. Rev. Phys. Chem.* **29**, 189-222 (1978).

[18] Peter, L. M., Wijayantha, K. G. U. & Tahir, A. A. Kinetics of light-driven oxygen evolution at α-Fe_2O_3 electrodes. *Faraday Discuss.* **155**, 309-322 (2012).

[19] Gelderman, K., Lee, L. & Donne, S. W. Flat-Band Potential of a Semiconductor: Using the Mott–Schottky Equation. *J. Chem. Educ.* **84**, 685-688 (2007).

[20] Kauranen, M. & Zayats, A. V. Nonlinear plasmonics. *Nature Photon.* **6**, 737-748 (2012).

[21] Shen, S., Lindley, S. A., Chen, X. & Zhang, J. Z. Hematite heterostructures for photoelectrochemical water splitting: rational materials design and charge carrier dynamics. *Energy Environ. Sci.* **9**, 2744-2775 (2016).

[22] Adireddy, S., Yao, Y., He, F. & Wiley, J. B. Rapid solvothermal fabrication of hexaniobate nanoscrolls. *Mater. Res. Bull.* **48**, 3236-3241 (2013).

[23] Kubacka, A., Fernandez-García, M. & Colon, G. Advanced Nanoarchitectures for Solar Photocatalytic Applications. *Chem. Rev.* **112**, 1555-1614 (2012).

[24] Bernardi, M., Mustafa, J., Neaton, J. B. & Louie, S. G. Theory and computation of hot carriers generated by surface plasmon polaritons in noble metals. *Nature Commun.* **6**, 7044 (2015).

[25] Khurgin, J. B. How to deal with the loss in plasmonics and metamaterials. *Nature Nanotech.* **10**, 2-6 (2015).

[26] Knight, M. W.et al. Embedding plasmonic nanostructure diodes enhances hot electron emission. *Nano Lett.* **13**, 1687-1692 (2013).

[27] Clavero, C. Plasmon-induced hot-electron generation at nanoparticle/metal-oxide interfaces for photovoltaic and photocatalytic devices. *Nature Photon.* **8**, 95-103 (2014).

[28] Furube, A., Du, L., Hara, K., Katoh, R. & Tachiya, M. Ultrafast Plasmon-Induced Electron Transfer from Gold Nanodots into TiO_2 Nanoparticles. *J. Am. Chem. Soc.* **129**, 14852-14853 (2012).
[29] Jiang, X. F. et al. Excitation nature of two-photon photoluminescence of gold nanorods and coupled gold nanoparticles studied by two-pulse emission modulation spectroscopy. *J. Phys. Chem. Lett.* **4**, 1634-1638 (2013).
[30] Valenti, M. et al. Hot Carrier Generation and Extraction of Plasmonic Alloy Nanoparticles. *ACS Photonics* **4**, 1146-1152 (2017).
[31] Li, J. T. et al. Solar hydrogen generation by a CdS-Au-TiO_2 sandwich nanorod array enhanced with Au nanoparticle as electron relay and plasmonic photosensitizer. *J. Am. Chem. Soc.* **136**, 8438-8449 (2014).
[32] Murphy, A. B. Efficiency of solar water splitting using semiconductor electrodes. *Int. J. Hydrogen Energy* **31**, 1999-2017 (2006).
